# Chemical reaction-transport model of oxidized diethylzinc based on quantum mechanics and computational fluid dynamics approaches†

**DOI:** 10.1039/c7ra11534b

**Published:** 2018-01-03

**Authors:** Jian Li, Hanlin Gan, Yifeng Xu, Chaoyang Wang, Feng Long Gu, Gang Wang

**Affiliations:** School of Electronics and Information Technology, Sun Yat-sen University Guangzhou 51000 China stswangg@mail.sysu.edu.cn; Key Laboratory of Theoretical Chemistry of Environment, Ministry of Education, School of Chemistry and Environment, South China Normal University Guangzhou 510006 China gu@scnu.edu.cn; State Key Laboratory of Optoelectronic Materials and Technologies Guangzhou 510275 China

## Abstract

We developed and studied a chemical reaction-transport model for the production of zinc oxide (ZnO) with diethylzinc (DEZn) and oxygen (O_2_). It was confirmed that a large number of ZnO particles were generated during the growth process by testing the internal particles of the cavity by X-ray diffraction. The formation of Zn_3_O_3_ in the gas phase reaction was simulated using density functional theory, and the effect of nucleation and formation of nanoparticles on the growth of the films was revealed. We also speculate that the adsorption of Zn-containing gas on the wall is the main route by which a ZnO film is formed. The mechanism calculated by quantum chemistry was applied in computational fluid dynamics (CFD) simulations using Fluent14.0 software, and the concentration distribution and gas reaction path of the reaction chamber were calculated and analyzed. Finally, a 9 gas phase reaction model and an 8 surface reaction model were established. Together with the transport model, a complete chemical reaction-transport reaction model was constructed for the ZnO–MOCVD cavity. The validity of the model was verified, and the optimum temperature range of DEZn and oxygen-stabilized growth of ZnO films was determined to be 673–873 K. Using the results of the chemical reaction transport model, the geometry and operation parameters of the reactor can be optimized to improve the characteristics of the epitaxial layer.

## Abstract 2

Chemical reaction-transport model of ZnO thin film deposited by DEZn and O_2_ in MOCVD.

## Graphical abstract

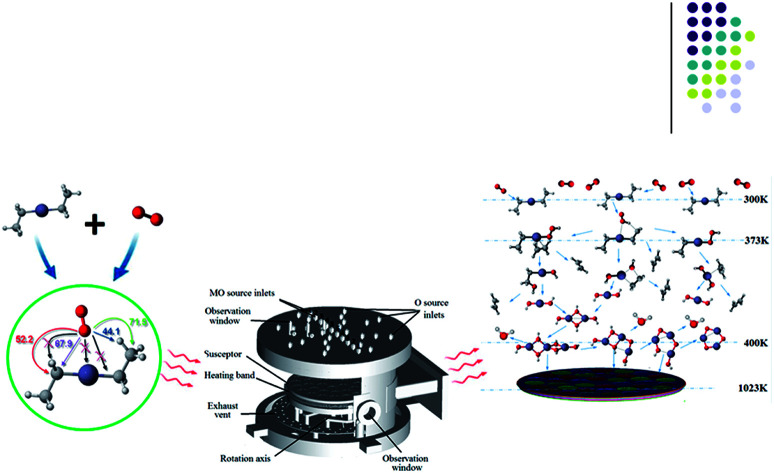

## Introduction

ZnO has attracted increasing attention in recent years as a new type of wide bandgap semiconductor material.^1–3^ ZnO has good physical properties: a direct bandgap band structure, room temperature bandgap of 3.37 eV, and exciton binding energy of 60 meV,^4^ which are the necessary conditions for high-efficiency ultraviolet light emission at room temperature. The use of light emitting diodes as blue-violet (LED) and UV laser (LD) has broad prospects for development for application as UV detectors, thin film transistors, spin field-effect transistors, and nanodevices.^5,6^ ZnO films can be prepared *via* many methods, including sputtering, pulsed-laser deposition (PLD), molecular beam epitaxy (MBE), sol–gel method, spray pyrolysis, and metal organic chemical vapor deposition (MOCVD). MOCVD is an important chemical process in the semiconductor industry that is used to produce thin film materials and high-tech devices because of this method's delivery of high quality films with a uniform and controllable growth source and rapid, large-area, and simultaneous growth. However, there are many complicated physical and chemical processes at work in MOCVD, and the reaction mechanism and growth kinetics are very complex.^7–11^ Mastering this technology to grow high-quality ZnO films requires a full understanding of the chemical reaction path that occurs during ZnO growth. The optimization of the reactor design is of great significance to the production of high-quality films.

Given the variety of substrate surfaces and the uncertain electronic structure of the solid surface, it is more challenging to obtain the parameters of the surface reaction rate than those of the gas phase reaction rate. At present, most of the chemical kinetic parameters of surface reactions are only numerical estimates. The mechanism of III–V compound,^12–21^ such as GaAs, InP, InGaAsP, GaN, and AlN, mainly focus on gas phase reactions. Theodoropoulos *et al.*^17^ expressed the kinetic parameters in terms of total bimolecular collision rates to represent the pre-exponential factors of the adsorption reaction when they studied the deposition of GaN thin films in MOCVD. Only a few of the kinetic parameters including the activation energy and the pre exponential factor used for gas phase reactions are experimentally obtained test values, and the rest are calculated or estimated by quantum chemical methods. In the case of InP growth, Theodoropoulos *et al.*^21^ took the wall reaction rate parameters (activation energy and pre-exponential factor) as adjustable parameters. These parameters were determined by fitting the experimental values of InP growth rate.

However, relatively few studies have been done on the compounds of the group II–VI.^22–27^ Dumont *et al.* used metal–organic chemical vapor deposition to oxidize DEZn and deposited a polycrystalline transparent semiconducting ZnO film.^22^ They found that the deposition process of thin films can be a complex multi-step oxidation process, consisting of a series of free radical reactions. Stanley and Schlegel studied the reaction process of DEZn and H_2_O and pointed out some of the probable intermediates and pathways for gas phase reactions involved in zinc oxide chemical vapor deposition.^23^ However, this study only considered the thermodynamics, and no chemical reaction mechanism gave a full path. Maejima *et al.* installed a Fourier transform infrared spectrometer (FTIR) on a ZnO MOCVD system.^27^ They used the DEZn and N_2_O reactions to grow the chemical gas phase reactions in the ZnO process and inferred the simple gas phase pre-reaction process between DEZn and N_2_O. So far, the kinetic parameters of the chemical mechanisms of the compounds of II–VI group are few and far between, which limits the understanding of the MOCVD reaction cavity flow and the growth of the films. Development of an understanding of chemistry and reaction mechanisms underlying MOCVD of ZnO is critical, therefore, to the fabrication of oxide-based devices.

In this study, a vertical rotating ZnO MOCVD reaction chamber was used as a prototype, and quantum chemical calculations were used to investigate the DEZn and O_2_ reaction to develop a chemical reaction pathway. The kinetics of the reaction system of gaseous ZnO and the chemical reaction path as well as the kinetic parameters were calculated by computational fluid dynamics (CFD) simulation as input parameters for constructing a chemical reaction-transport model for DEZn oxidation. The deposition rate behavior of ZnO with temperature is in agreement with that reported in literature. Moreover, further studies were conducted on the MOCVD reaction chamber components and chemical reactions, and the growth process of ZnO in the ZnO–MOCVD reaction cavity was determined. The proposed model may become a useful tool for reactor design, optimization, and scale-up of ZnO MOCVD.

## Reaction paths and kinetic parameters

**Fig. 1:**
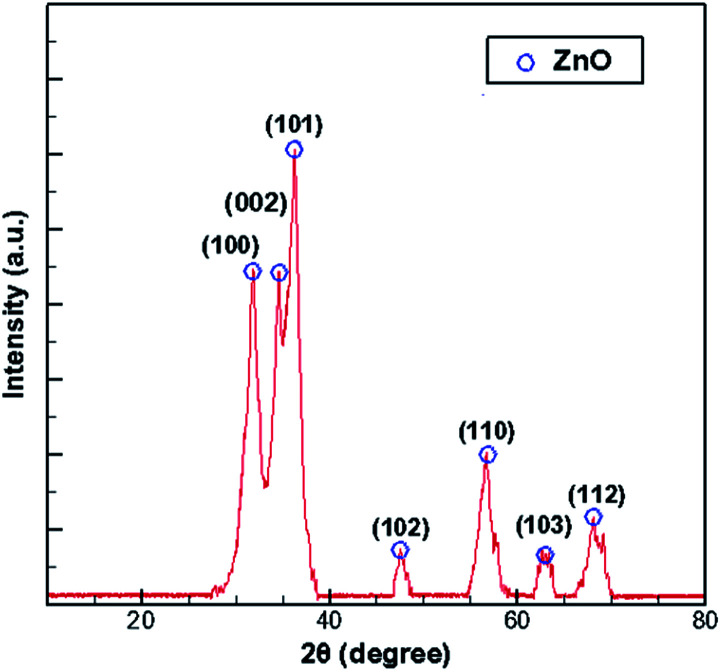
Nanoparticles in the ZnO–MOCVD reaction chamber and the XRD spectra of the ZnO particles.

In the process of growing ZnO in MOCVD using DEZn and O_2_, adjusted according to different process parameters, the general set of chamber pressure range is 6–20 tor, the substrate temperature is 723–823 K, the total flow control between 15–30 slm, the growth time of 45–70 min, nano deposition thickness is about 200–300 nm. During maintenance of the ZnO–MOCVD reactor cavity, many solid particles could be seen in the cavity. They are mainly covered on the base of the cavity, with an average thickness of about 2 mm. The solid particles were tested by XRD and identified as ZnO solid particles, as shown in Fig. 1. The XRD pattern shows 7 peaks at 31.8°, 34.4°, 36.3°, 47.5°,56.6°, 62.9°, 68.0°, indexed to (100), (002), (101), (102), (110), (103), (112) planes of the ZnO crystal given by the standard data file (JCPDS card no. 36-1451).^28^ According to the Scherrer equation:
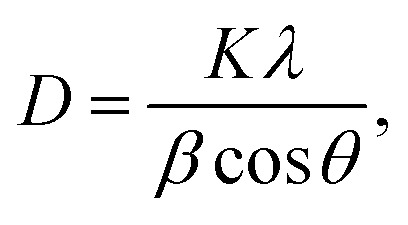
where *D* is the grain diameter (nm), *K* is a constant, *λ* is X-ray wavelength, *β* is the half peak width of diffraction angle, *θ* is the Prague diffraction angle. The average diameter of the particles with 7 peaks is 12.65 nm. The nanoparticle definition designated by the National Nanotechnology Initiative has been adopted by the American National Standards Institute as particles with all dimensions between 1 nm and 100 nm.^29,30^ Therefore, we believe that ZnO nanoparticles can be formed by a gas phase reaction. The study by Chen^31^ indicated that a trimer of zinc oxide (Zn_3_O_3_) may be formed as a nanoparticle precursor. Oligomers form from the trimer and can nucleate to grow into nanoparticles. In the process of generating the trimer, the Zn-containing gas component is adsorbed on the substrate surface to form a ZnO film.

The B3LYP/6-311G(d)^32,33^ level of theory as implemented in the GAUSSIAN 09 package^34^ has been utilized in the full geometry optimizations of reactants, intermediates, products, and transition states (TS). Analytical vibrational frequency calculations have been performed for all optimized compounds on the same level of theory. Exactly, the stationary points (reactants, intermediates, and products) are characterized by zero imaginary vibrations while exactly one imaginary vibration characterizes transition structures. Each TS structure that connects two relevant stationary points is confirmed by the intrinsic reaction coordinate (IRC)^35,36^ calculations. For the structures of every point along each IRC calculation, single-point calculations in singlet state are performed to locate the spin conversion points where singlet and triplet potential energy surfaces (PES) crossing. The structures whose energies in triplet state nearly equal to that of the singlet state are the potential spin conversion points (CP), and they are the initial structures to input to geometry optimize on the same level using the modified version of Harvey's minimal energy crossing points (MECP) program, namely the sobMECP^37,38^ program. In order to obtain more accurate electronic energies during dehydration of zinc hydroxide, single-point calculations were performed at the CCSD(T)/6-311+G(d,p) level of theory for the B3LYP/6-311G(d)-optimized geometries. The optimized compounds are illustrated using CYLview^39^ program to depict the electronic energy profile of the dominant reaction path.

### Gas phase reactions

**Fig. 2:**
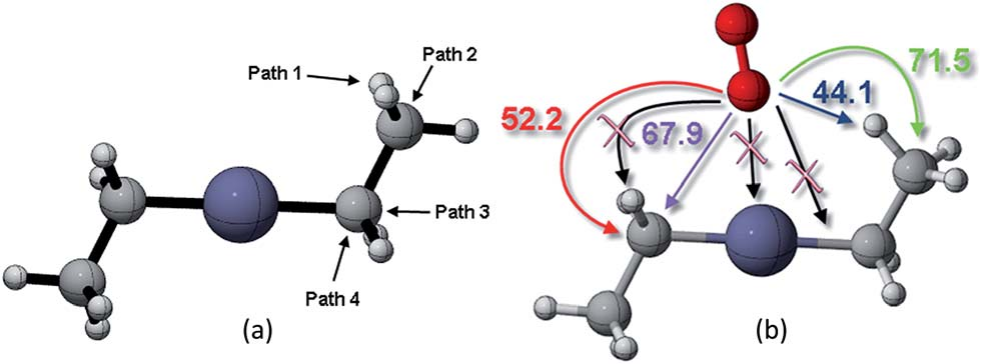
(a) Different reaction paths for oxygen to attack on DEZn and their corresponding (b) energy barriers scenarios.

This study analyzed four different pathways for oxygen attack on DEZn: (1) O_2_ abstracts methyl H, (2) O_2_ attacks on methyl C, (3) O_2_ attacks on methylene C to break Zn–C bond, (4) O_2_ attacks on methylene C to break the C–C bond, as shown in Fig. 2. The corresponding energy barriers for the attack scheme are determined according to the different attack pathways, as shown in Fig. 2(b). Kinetically, the main reaction path is methyl H abstraction.

**Fig. 3:**
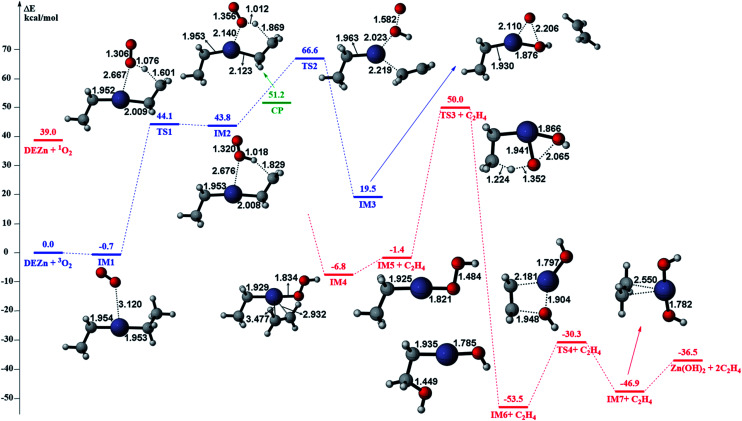
The potential energy surface (PES) profile of the favourable reaction paths for the reaction Zn(C_2_H_5_)_2_ + 3O_2_ → Zn(OH)_2_ + 2C_2_H_4_. Energies are in kcal mol^−1^ and distances are in angstrom. The reaction paths on triplet and singlet PES are illustrated in blue and red line respectively. The spin conversion point is illustrated in green line and denoted as CP.

**Fig. 4:**
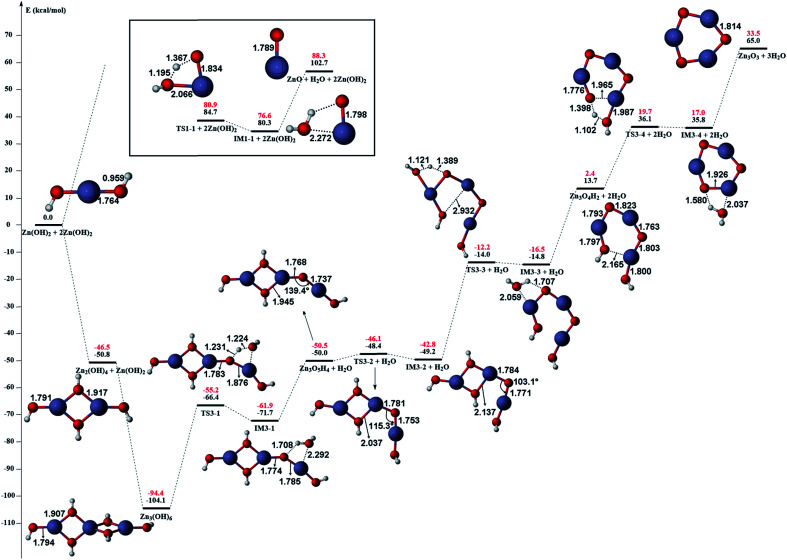
The potential energy surface (PES) profile of zinc hydroxide dehydration computed at B3LYP/6-311G(d) (black) and CCSD(T)/6-311+G(d,p) (red) levels of theory. The relative electronic energies are given in kcal mol^−1^ while the bond lengths are given in angstrom.

The gas phase reaction paths and the energy barriers are shown in Fig. 3 and 4. (The spin conversion point is located in the path from IM2 to TS2. That is to say, during the process for the hydroxyl group of ˙OOH to attack the zinc atom and cleave the Zn–C bond, the reaction is suggested to occur through a spin conversion point from triplet to singlet PES to generate IM4 on singlet PES instead of leading to TS2). Based on the formation process of Zn_3_O_3_, we identified nine gas phase reactions and calculated the kinetic parameters of the reaction kinetics of G1–G9, *i.e.*, the prefactor and activation energy, using the parameter calculation method,^17,18,40^ as shown in Table 1. The reaction of DEZn and O_2_ is similar to that of DEZn and H_2_O, and the reaction model of ZnO is similar to that in reference.^23^ That is, the process involves forming Zn(OH)_2_, then producing oligomers, and finally oligomer dehydration. In the model, we consider the complexation of triplet oxygen on the zinc site of DEZn (G1), methyl H abstraction by triplet oxygen (G2), spin conversion during the process for hydroxyl group of HOO˙ to attack zinc atom (G3), intramolecular proton transfer of HOOZnC_2_H_5_ (G4), and hydroxyl group transfer of HOCH_2_CH_2_ZnOH leading to Zn(OH)_2_ (G5). The generation of ZnO by Zn(OH)_2_ alone requires an energy of 80.9 kcal mol^−1^, but the formation of oligomers makes ZnO more accessible (G6–G9), requiring only 39.2 kcal mol^−1^. With increasing temperature, polymers aggregate into larger nanoparticles that are sent out with exhaust gas, resulting in lower utilization of gas. In the gas phase model, the chemical reaction paths in the ZnO–MOCVD reaction chamber is fully reflected by an addition reaction, adduct decomposition reaction, and polymerization reaction. At the same time, the simplicity of the model is maintained in order to reduce the computational cost.

**Table 1:** Gas phase reaction pathways and kinetic parameters from DEZn and O_2_^a^

NO	Reactions	*A*	*E*_a_	*n*
G1	Zn(CH_2_CH_3_)_2_ + O_2_ → Zn(CH_2_CH_3_)_2_·O_2_	Coll	0	0
G2	Zn(CH_2_CH_3_)_2_·O_2_ → ˙CH_2_CH_2_ZnC_2_H_5_·HOO˙	3.34 × 10^12^	44.8	0
G3	˙CH_2_CH_2_ZnC_2_H_5_·HOO˙ → HOOZnC_2_H_5_ + C_2_H_4_	Coll	7.4	0
G4	HOOZnC_2_H_5_ → HOCH_2_CH_2_ZnOH	1.36 × 10^12^	51.4	0
G5	HOCH_2_CH_2_ZnOH → Zn(OH)_2_ + C_2_H_4_	1.29 × 10^14^	23.2	0
G6	3Zn(OH)_2_ → Zn_3_(OH)_6_	Coll	0	0
G7	Zn_3_(OH)_6_ → Zn_3_O_5_H_4_ + H_2_O	2.42 × 10^13^	39.2	0
G8	Zn_3_O_5_H_4_ → Zn_3_O_4_H_2_ + H_2_O	1.67 × 10^12^	30.6	0
G9	Zn_3_O_4_H_2_ → Zn_3_O_3_ + H_2_O	1.08 × 10^12^	17.3	0

a Activation energies are in kcal mol^−1^ and pre-exponentials are in s^−1^.

The accuracy of the activation energies would make a great difference in CFD calculations. Thereby, the geometries optimized at the B3LYP/6-311G(d) level were used to carry out CCSD(T) single-point energy calculations with 6-311+G(d,p) basis set. The electronic energy of the CP should be noted. Because the geometry of CP has been optimized at the B3LYP/6-311G(d) level, the electronic energy of singlet state for CP structure is nearly equal to the triplet one at the B3LYP/6-311G(d) level. However, there is a differential between the electronic energies from CCSD(T) single-point calculations of the CP singlet and triplet state. This could result in the difficulty in calculating the energy barriers of oxidation of DEZn at the CCSD(T)/6-311+G(d,p) level. Therefore, in Fig. 3, only the barriers at the B3LYP/6-311G(d) level could be obtained and they are applied in the followed CFD calculation. For the dehydration of zinc hydroxide in gas phase, the reactions only occur on the singlet potential surface, so the CCSD(T)/6-311+G(d,p)//B3LYP/6-311G(d) activation energies would be obtained (Fig. 4) and we use them in the CFD model. The relative energies in Fig. 4 reveal that the B3LYP/6-311G(d) energies overestimate the thermodynamical unfavorability. In other words, it could be inferred from the CCSD(T)/6-311+G(d,p) results that the ZnO solid particles in the cavity could be produced at lower temperature relative to the B3LYP/6-311G(d) results.

### Surface reactions

In this study, a simplified surface reaction kinetic model is used to describe the surface growth of the film, as shown in Table 2. The principle and simplification of the model are as follows: (1) the growth rate does not depend on the number of O_2_ but depends only on the number of Zn-containing species that arrive at the substrate surface. (2) Considering that the energy barrier of the gas phase product generated after Zn(CH_2_CH_3_)_2_·O_2_ is 44.8 kcal mol^−1^ (G2).

**Table 2:** Surface reaction pathways and kinetic parameters for ZnO growth^a^

No.	Reaction	*A*	*E*_a_
S1	Zn(CH_2_CH3)_2_·O_2_ → ZnO + 2C_2_H_4_ + H_2_0	*S* = 1	9.56
S2	˙CH_2_CH_2_ZnC_2_H_5_·HOO˙ → ZnO + 2C_2_H_4_ + H_2_O	*S* = 1	0
S3	HOOZnC_2_H_5_ → ZnO + 2C_2_H_4_ + H_2_O	*S* = 1	0
S4	HOCH_2_CH_2_ZnOH → ZnO + 2C_2_H_4_ + H_2_O	*S* = 1	0
S5	Zn(OH)_2_ → ZnO + H_2_O	*S* = 1	0
S6	Zn_3_(OH)_6_ → 3ZnO + 3H_2_O	*S* = 1	0
S7	Zn_3_O_5_H_4_ → 3ZnO + 2H_2_O	*S* = 1	0
S8	Zn_3_O_4_H_2_ → 3ZnO + H_2_O	*S* = 1	0

a *S* = 1 denotes a unity sticking coefficient at zero coverage.^17,18^ Activation energies are in kcal mol^−1^.

However, the adsorption reaction on the wall is lower in energy than the reaction temperature of the gas phase. Therefore, we use the activation energy of S1 as an adjustable parameter,^21^ which is determined by the ZnO growth rate and the literature value fitting method, and the adsorption energy of other surface reactions is 0. (3) The surface adsorption and reactions of all gas containing species except Zn_3_O_3_ are considered in the model.

## CFD model and boundary conditions

**Fig. 5:**
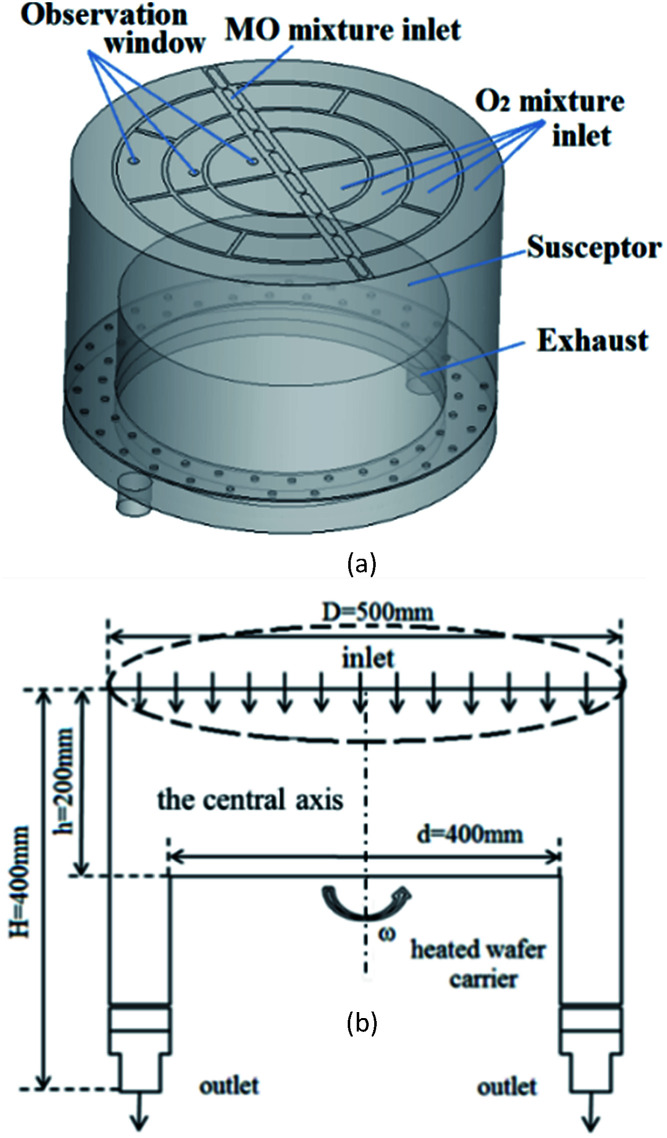
(a) Simulation model diagram of ZnO–MOCVD reaction chamber (b) two-dimensional flow diagram.

We used DEZn and O_2_ sources to model ZnO growth. The gases were injected from the showerhead to the chamber and were carried by Ar. A schematic diagram of the model is shown in Fig. 5(a), and the flow diagram is shown in Fig. 5(b). The MOCVD growth process of ZnO was performed in a low-pressure reactor with a laminar flow. The mixed gas can be treated as an ideal, incompressible gas mixture. The system was presumed to be in a steady state. The following assumptions and boundary conditions were made in this study, under the premise that they have little effect on the main results:

(1) The MO and Ar carrier gas mixture enters the reactor through the central MO mixture inlet, and O_2_ mixture inlets are distributed on both sides in this model. Among them, the content of Ar is 15 450 sccm, the DEZn content is 90 sccm, and the O_2_ content is 550 sccm.

(2) The graphite disc was assumed to have good thermal conductivity, and its temperature was assumed to be consistent with that of the substrate (423–1223 K). The rotation speed of the susceptor was set to 750 rpm, and the internal pressure of the cavity is 6.1 Torr.

(3) At the exhaust, the pressure-outlet boundary condition was applied, *i.e.*, a static pressure of 0 Pa at the outlet.

## Verification and analysis of calculation results

**Fig. 6:**
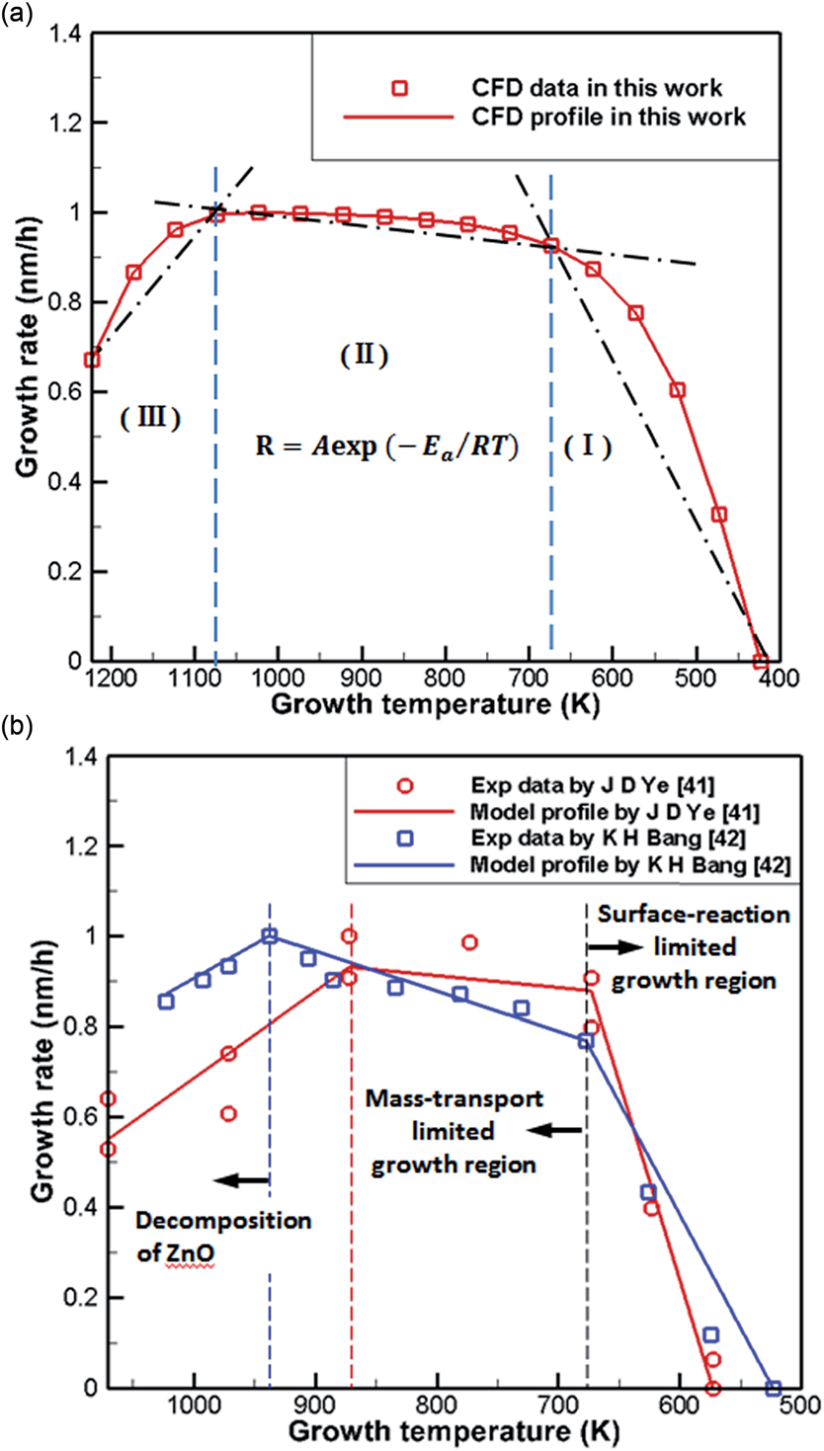
(a) Normalization results of CFD simulations (b) Arrhenius plots of the growth rate *versus* reciprocal temperature in literature.

Fig. 6(a) shows normalization results from the literature^41,42^ and CFD simulations of the growth rate of ZnO generated by the reaction of DEZn with O_2_ at different temperatures. It can be seen from the processed data that the numerical simulation of CFD is in good agreement consistent with the grow trend in Fig. 6(b), proving the feasibility of the current chemical reaction-transport model of DEZn with O_2_. Fig. 6 shows three distinct regimes: the growth rate of ZnO increases rapidly with the growth temperature below 673 K (regime I). The main reason for this increase is that the growth rate is affected by the surface reaction kinetics at a lower temperature. The low-temperature region (*T* < 673 K) and the numerical fitting of the literature are better, and the rationality of the S1 activation energy is proved. Above 1073 K (regime III), the rate of formation of nanoparticles exceeds the deposition rate of the films when the temperature is increased. Excessive Zn_3_O_3_ formation leads to a waste of resources, resulting in a decrease in the deposition rate of ZnO. At 673–1073 K (regime II), the deposition rate changes relatively smoothly, and the growth rate is considered to be controlled by mass transport. In this interval, the growth efficiency is high, and the growth rate is less sensitive to the temperature.

**Fig. 7:**
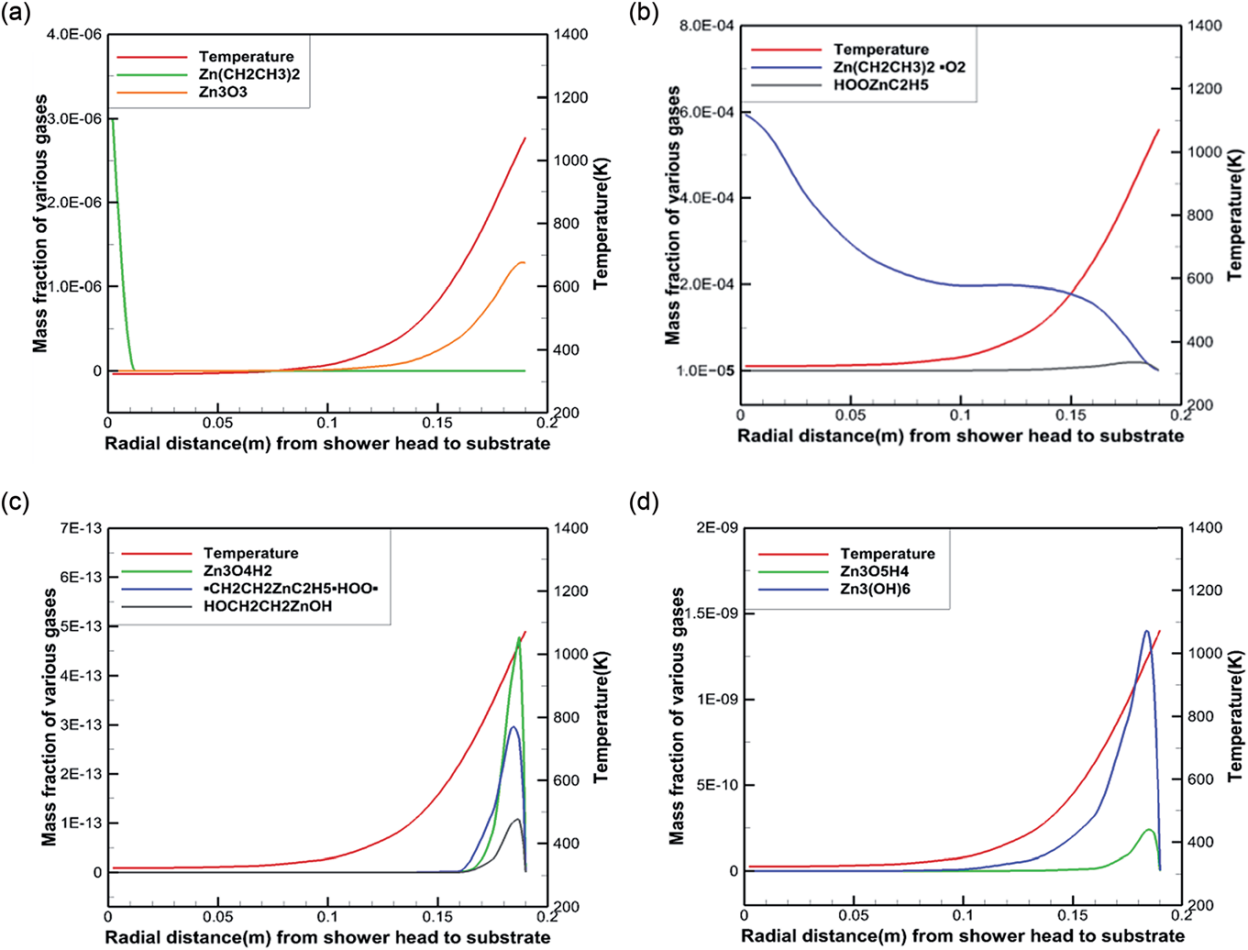
Temperatures and mass fractions of the main component along the chamber radius.

To better reveal the chemical reaction state inside the cavity, we analyzed the ZnO deposition rate, which begins to decline at 1073 K. Fig. 7 depicts the mass fraction of different Zn sources in the gas mixture on the central axis of the cavity at 1073 K. It can be concluded that the mass fraction contour can provide a basis to better understand the mass transfer and reaction mechanisms in the reactor. At 300 K, DEZn and O_2_ react violently with each other to form Zn(CH_2_CH_3_)_2_·O_2_ after the formation of mixed complexes, as shown in Fig. 7(a) and (b). In the cavity, Zn(CH_2_CH_3_)_2_·O_2_ is the most abundant species. In the cavity, Zn(CH_2_CH_3_)_2_·O_2_ is the most abundant species, which decomposes in the reaction chamber, thus, reaction G2 is the decisive step of the whole reaction. At even higher temperatures, Zn(CH_2_CH_3_)_2_·O_2_ continually decomposes. At 373 K, HOOZnC_2_H_5_ begins to be generated. At 400 K, the simultaneous occurrence of Zn_3_ (OH)_6_ and Zn_3_O_3_ prove that both Zn_3_O_5_H_4_ and Zn_3_O_4_H_2_ have been completely reacted at this point, as shown in Fig. 7(c) and (d). However, at higher temperatures, more Zn_3_O_3_ is formed on the substrate surface, which affects the deposition rate and film quality.

**Fig. 8:**
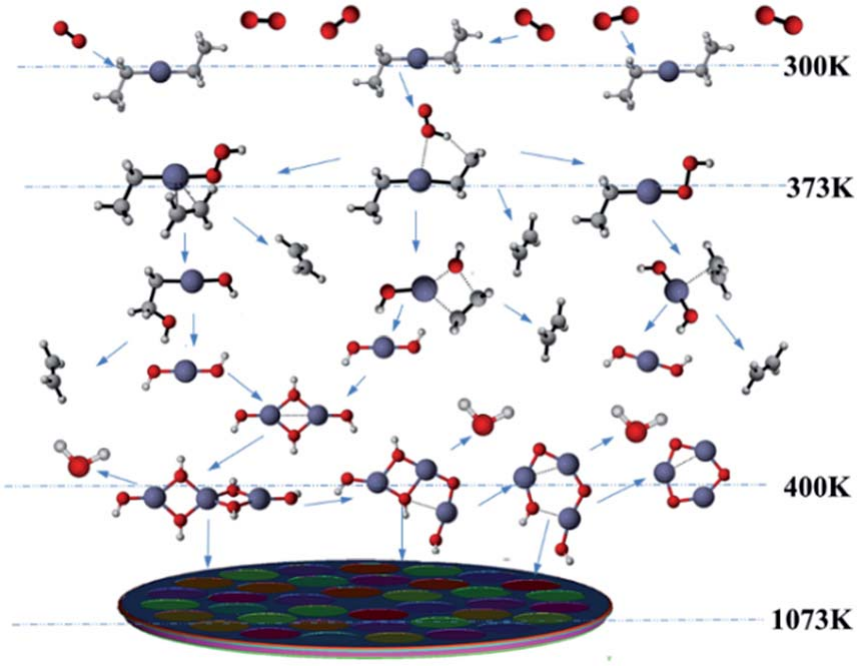
Schematic illustration of the chemical reactions in the vapor phase in the MOCVD chamber.

Based on our results, the reaction path of DEZn oxidization in this MOCVD reaction chamber is shown at 1073 K in Fig. 8. When the temperature is greater than 400 K, Zn_3_O_3_ is produced within the cavity, leading to nanoparticle formation. This process can occur alongside the growth of the film.

The difference between the present results and previous studies is that the decomposition reaction does not just occur in regime III. Only Zn_3_O_3_ is generated in large amounts in this temperature region, resulting in a decrease in the deposition rate of ZnO. Thus, we observed the content of Zn_3_O_3_ at 823–1173 K to better determine the growth range, as shown in Table 3. Little Zn_3_O_3_ (mass fractions below 1 × 10^−10^) was produced below 873 K. For this reason, we believe that the optimum growth temperature range of ZnO from DEZn and O_2_ is 673–873 K.

**Table 3:** Mass fraction of Zn_3_O_3_ at the substrate surface at different temperatures

	*T*							
	823 K	873 K	923 K	973 K	1023 K	1073 K	1123 K	1173 K
Mass fractions	8.78 × 10^−13^	5.60 × 10^−11^	1.62 × 10^−9^	2.62 × 10^−8^	2.63 × 10^−7^	1.27 × 10^−6^	6.59 × 10^−6^	1.98 × 10^−5^

## Conclusion

We proposed a chemical reaction-transport model for ZnO production by the reaction of DEZn with O_2_ during MOCVD. The kinetic parameters of the reaction steps were obtained using quantum chemistry calculations. Based on these data, the reaction and ZnO deposition processes in a MOCVD system were studied using CFD software. The following conclusions can be drawn.

(1) The nanoparticles formed inside the ZnO–MOCVD cavity were analyzed by XRD, and the main component of the nanoparticles was ZnO. Therefore, it is speculated that there is a corresponding path of ZnO formation from oligomers during the gas phase reaction.

(2) According to the different routes of oxygen attack on DEZn, the chemical reaction path is determined by the minimum energy principle, that is, the main reaction path is O_2_ capture by methyl H. Zn_3_O_3_ was identified as the initiator for nanoparticles to form oligomers that then nucleated into ZnO. Finally, the gas phase reaction was determined to involve the following conversion pathway: DEZn to Zn(OH)_2_ to Zn_3_O_3_.

(3) The high temperature required by gas phase reaction means that the gas phase component containing Zn in the G1 path is adsorbed on the surface of the substrate during the low-temperature process, leading predominantly to film formation. The activation energy of S1 chemical reaction is estimated, and the activation energy of the equation is 9.56 kcal mol^−1^, the tendency of deposition rate of substrate film calculated by CFD is consistent with the experimental in the literature.

(4) The chemical kinetic parameters were integrated into the CFD analysis to validate the experimental observations in a MOCVD chamber. The effect of the temperature distribution on the ZnO film growth rate was discussed. At low temperatures (<673 K), the growth rate of the films is exponential and controlled by the surface reaction kinetics. In the region of 673–1073 K, the film growth rate is stable and mainly controlled by the gas flow rate. When the temperature is higher than 1073 K, the growth rate of the thin films is reduced, possibly due to the formation of nanoparticles from the gas phase polymer.

(5) The analysis of the Zn_3_O_3_ nanoparticles precursor at different temperatures shows that the amount of Zn_3_O_3_ produced below 873 K is very low, the mass fraction of Zn_3_O_3_ is 1 × 10^−5^ of Zn(CH_2_CH_3_)_2_·O_2_ and 1 × 10^−2^ of HOOZnC_2_H_5_. Therefore, to prevent pre-reaction, the optimum DEZn and O_2_ reactions yield ZnO film material with a temperature of 673–873 K.

## Experimental

XRD spectra were recorded using a D8 Advance X-ray diffractometer (Bruker, Germany). The high precision goniometer guaranteed that the errors in the peak position and standard peak position of each diffraction peak in the full spectrum range was not more than 0.01 degrees

## Conflicts of interest

There are no conflicts to declare.

## Supplementary Material

RA-008-C7RA11534B-s001
