# Supplementary material for: Chemical reaction-transport model of oxidized diethylzinc based on quantum mechanics and computational fluid dynamics approaches
Source: RSC Adv. 2018 Jan 3;8(2):1116–23. doi: 10.1039/c7ra11534b (PMC9077139; doi:10.1039/c7ra11534b)
Supplement: RA-008-C7RA11534B-s001 [file RA-008-C7RA11534B-s001.pdf]

Electronic Supplementary Material (ESI) for RSC Advances.
This journal is © The Royal Society of Chemistry 2017

Supporting Information for

Chemical reaction-transport model of oxidized diethylzinc based on quantum mechanics and computational fluid dynamics approaches

Jian Li [a], Hanlin Gan [b], Yifeng Xu[a], Chaoyang Wang [b], FengLong Gu [*b], Gang Wang[*a,c]

[a]School of Electronics and Information Technology, Sun Yat-sen University, Guangzhou 51000, China
[b]Key Laboratory of Theoretical Chemistry of Environment, Ministry of Education, School of Chemistry and Environment, South China Normal University, Guangzhou 510006, China
[c]State Key Laboratory of Optoelectronic Materials and Technologies, Guangzhou 510275, China

number of pages: 29

number of tables: 29

number of figures: 5

Geometries Optimized at B3LYP/6-311G(d)

Table S1

Structure: IM1 -2088.1551506 Hartree

0 3

Zn	0.440591	-0.527181	-0.190144
C	1.729902	0.929990	-0.008433
H	1.680995	1.557764	-0.906342
H	1.412130	1.580602	0.815433
C	3.180082	0.475280	0.223780
H	3.869925	1.322110	0.320351
H	3.282975	-0.121131	1.136379
H	3.553280	-0.143460	-0.599007
O	-1.574673	1.768838	-0.821649
H	-2.674399	-0.912541	0.124137
O	-1.493141	2.921017	-0.475886
C	-0.827403	-2.004281	-0.348288
H	-0.336373	-2.929402	-0.023331
H	-1.060261	-2.157981	-1.408869
C	-2.132799	-1.809552	0.441202
H	-2.820599	-2.654732	0.319247
H	-1.951197	-1.703485	1.515957

Table S2

Structure: TS1 **-2088.0837547 Hartree**

0 3

**Zn**	**0.497572**	**-0.339560**	**0.009114**
**C**	**1.985624**	**0.923908**	**0.010382**
**H**	**1.855485**	**1.586115**	**-0.852449**
**H**	**1.875127**	**1.567308**	**0.890156**
**C**	**3.384805**	**0.291962**	**-0.011164**
**H**	**4.176179**	**1.050592**	**-0.009534**
**H**	**3.561110**	**-0.349923**	**0.858077**
**H**	**3.543872**	**-0.328047**	**-0.899387**
**O**	**-1.701096**	**1.163881**	**-0.133862**
**H**	**-2.173044**	**0.198414**	**-0.085747**
**O**	**-2.570608**	**2.111784**	**0.091263**
**C**	**-0.867165**	**-1.813201**	**0.022434**
**H**	**-0.624188**	**-2.377454**	**0. 929204**
**H**	**-0.601826**	**-2.427484**	**-0.844074**
**C**	**-2.278503**	**-1.396781**	**-0.006511**
**H**	**-2.852889**	**-1.532477**	**-0.919981**
**H**	**-2.861924**	**-1.440875**	**0.910276**

Table S3

Structure: IM2 **-2088.0841882 Hartree**

0 3

**Zn**	**0.503426**	**-0.383902**	**-0.037 249**
**C**	**1.967634**	**0.907620**	**-0.059617**
**H**	**1.866909**	**1.505174**	**-0.972138**
**H**	**1.805069**	**1.608651**	**0.766308**
**C**	**3.375974**	**0.302483**	**0.026647**
**H**	**4.154476**	**1.074189**	**0.009889**
**H**	**3.522117**	**-0.273252**	**0.946364**
**H**	**3.585576**	**-0.375445**	**-0.807155**
**O**	**-1.651411**	**1.189112**	**-0.250779**
**H**	**-2.157526**	**0.309714**	**-0.170342**
**O**	**-2.291681**	**2.106786**	**0.450278**
**C**	**-0.830067**	**-1.885052**	**0.008526**
**H**	**-0.568767**	**-2.423761**	**0.926317**
**H**	**-0.552942**	**-2.513258**	**-0.844471**
**C**	**-2.247 200**	**-1.511539**	**-0.022129**
**H**	**-2.823893**	**-1.624349**	**-0.935662**
**H**	**-2.820381**	**-1.468137**	**0.899530**

Table S4

Structure: CP **-2088.0724 Hartree**

Spin Conversion Point

Zn	0.270790	-0.007678	-0.052795
C	1.925606	1.036560	0.004801
H	1.891330	1.724980	-0.846423
H	1.876915	1.672783	0.894816
C	3.231014	0.233301	-0.006046
H	4.112731	0.884395	0.020062
H	3.307629	-0.437023	0.855884
H	3.324790	-0.389328	-0.901410
O	-1.614833	0.965561	-0.332743
H	-2.268293	0.244809	-0.055497
O	-1.918626	2.156097	0.241325
C	-0.877485	-1.791691	0.027806
H	-0.476797	-2.186332	0.964334
H	-0.450884	-2.291657	-0.8446 94
C	-2.281956	-1.623781	-0.000674
H	-2.840873	-1.705371	-0.926719
H	-2.862527	-1.597463	0.916171

Table S5

Structure: TS2 **-2088.0478947 Hartree**

0 3

**Zn**	**0.310620**	**-0.098699**	**0.030593**
**C**	**2.143500**	**-0.801348**	**0.070543**
**H**	**2.282223**	**-1.323227**	**1.024001**
**H**	**2.221497**	**-1.577060**	**-0.699238**
**C**	**3.252928**	**0.242448**	**-0.120503**
**H**	**4.250630**	**-0.210854**	**-0.084843**
**H**	**3.174377**	**0.756911**	**-1.083750**
**H**	**3.229772**	**1.015892**	**0.654009**
**O**	**-1.536018**	**-0.924689**	**-0.000699**
**H**	**-2.188428**	**-0.208515**	**-0.022500**
**O**	**-2.496700**	**-2.181261**	**-0.014360**
**C**	**-0.713210**	**1.869823**	**0.000967**
**H**	**-0.156567**	**2.167167**	**-0.890587**
**H**	**-0.215470**	**2.166375**	**0.926910**
**C**	**-2.091185**	**2.105310**	**-0.043729**
**H**	**-2.683742**	**2.177767**	**0. 861659**
**H**	**-2.624232**	**2.176513**	**-0.985424**

Table S6

Structure: IM3 **-2088.1229398 Hartree**

0 3

Zn	-1.060903	-0.060194	-0.366407
C	-2.560333	-1.275761	-0.371040
H	-2.703076	-1.631825	-1.396944
H	-2.281190	-2.163370	0.206516
C	-3.873562	-0.687092	0.164783
H	-4.688288	-1.419150	0.131824
H	-3.783632	-0.357222	1.204320
H	-4.200892	0.181092	-0.414947
O	-0.139712	1.574117	-0.333707
H	0.523674	1.816126	0.322582
O	1.033443	-0.286963	-0.494362
C	2.491579	3.446389	0.161917
H	2.652193	4.418774	0.618553
H	1.841342	3.417270	-0.706416
C	3.060626	2.342740	0.638612
H	2.898489	1.374313	0.175594
H	3.713515	2.368309	1.506626

Table S7

Structure: IM4 **-2088.1649436 Hartree**

0 1

Zn	0.110728	0.761479	0.390764
C	1.964254	0.598594	-0.117766
H	1.991776	0.360891	-1.186224
H	2.443719	1.577460	-0.014502
C	2.748460	-0.458341	0.672108
H	3.785279	-0.534567	0.325443
H	2.786896	-0.229230	1.741614
H	2.305210	-1.453476	0.573515
O	-2.276306	1.389070	-0.279904
H	-3.137177	1.661990	0.057694
O	-1.583538	1.108846	1.000990
C	-0.434704	-2.489125	-0.717786
H	0.216832	-3.213063	-0.237249
H	-0.328896	-2.389457	-1.794163
C	-1.315044	-1.766079	-0.030206
H	-1.982741	-1.053255	-0.502168
H	-1.432125	-1.878487	1.042982

Table S8

Structure: IM5 **-2009.5493196 Hartree**

0 1

Zn	-0.079120	-0.017705	0.009608
C	1.712747	0.686231	0.018090
H	1.800726	1.395363	-0.811263
H	1.842325	1.276646	0.930706
C	2.816063	-0.378224	-0.081100
H	3.813394	0.075191	-0.074864
H	2.783300	-1.085629	0.753008
H	2.740145	-0.966772	-1.000306
O	-2.542241	0.500140	-0.068821
H	-3.429786	0.125026	-0.074247
O	-1.746071	-0.750764	0.001759

Table S9

Structure: TS3 **-2009.4674813 Hartree**

0 1

Zn	0.334359	-0.732788	0.158109
C	-1.624205	-0.814778	0.054169
C	-1.913667	0.659907	-0.033925
H	-2.041695	-1.272464	0.951332
H	-1.961535	-1.365338	-0.824543
H	-0.880504	1.302794	0.096064
H	-2.269276	1.001273	-1.006011
H	-2.515387	1.067881	0.779176
O	2.079795	-0.081155	0.266001
O	0.465314	1.203387	0.178497
H	2.464619	0.272223	-0.542359

Table S10

Structure: IM6 **-2009.6324133 Hartree**

0 1

**Zn**	**0.429818**	**-0.422676**	**-0.139659**
**C**	**-1.493571**	**-0.501252**	**0.054222**
**C**	**-1.935442**	**0.948488**	**-0.057246**
**H**	**-1.747647**	**-0.916997**	**1.033023**
**H**	**-1.974458**	**-1.124017**	**-0.703476**
**H**	**-1.052864**	**2.689795**	**0.350995**
**H**	**-2.209424**	**1.193816**	**-1.091193**
**H**	**-2.793114**	**1.182384**	**0.586257**
**O**	**2.201211**	**-0.456874**	**-0.355225**
**O**	**-0.802841**	**1.758707**	**0.343715**
**H**	**2.676524**	**0.357974**	**-0.180147**

Table S11

Structure: TS4 **-2009.5953428 Hartree**

0 1

**Zn**	**-0.410804**	**0.595272**	**-0.011026**
**C**	**1.008386**	**-1.059286**	**-0.064920**
**C**	**2.119256**	**-0.232269**	**0.096389**
**H**	**0.855663**	**-1.521291**	**-1.036814**
**H**	**0.689451**	**-1.654658**	**0.787153**
**H**	**1.577126**	**2.386620**	**0.338323**
**H**	**2.572611**	**-0.099000**	**1.070394**
**H**	**2.752778**	**0.019930**	**-0.743185**
**O**	**-2.205380**	**0.689326**	**0.009149**
**O**	**1.254125**	**1.512809**	**0.086638**
**H**	**-2.687746**	**-0.082606**	**-0.292877**

Table S12

Structure: IM7 **-2009.6218768 Hartree**

0 1

Zn	-0.259355	0.206256	-0.017983
C	1.448915	-1.610732	-0.548011
C	1.788041	-1.153789	0.659597
H	1.853799	-1.162544	-1.449222
H	0.748797	-2.432076	-0.658542
H	0.352495	2.344197	-0.998493
H	1.371920	-1.592470	1.560368
H	2.476922	-0.322940	0.769684
O	-1.394716	-0.943267	0.735952
O	0.720735	1.487136	-0.778006
H	-2.309978	-0.714752	0.905791

Table S13

Structure: $Zn(OH)_2$ **-1930.9983068 Hartree**

0 1

Zn	-0.723842	0.302268	0.075846
O	0.386204	1.668943	0.189316
O	-1.821264	-1.073931	-0.045107
H	-1.930916	-1.626685	0.731380
H	0.279773	2. 383824	-0.441642

Table S14

Structure: TS1-1 **-1930.8633619 Hartree**

0 1

Zn	0.365858	-0.525001	-0.029107
O	0. 758338	1.266175	0.010229
O	-1.498238	0.364656	-0.075747
H	-2.151492	0.522460	0.610165
H	-0.603302	1.155816	-0.033484

Table S15

Structure: IM1-1 **-1930.8702615 Hartree**

0 1

**Zn**	**0.483417**	**-0.292404**	**0.004374**
**O**	**0.169196**	**1.478274**	**0.016149**
**O**	**-1.787068**	**-0.315848**	**-0.073126**
**H**	**-2.570057**	**-0.402595**	**0.477726**
**H**	**-1.484213**	**0.631408**	**-0.064139**

Table S16

Structure: ZnO **-1854.4006799 Hartree**

0 1

**Zn**	**0.484501**	**-0.369052**	**0.004135**
**O**	**0.417367**	**1.418522**	**0.007797**

Table S17

Structure: $Zn_2(OH)_4$ **-3862.0775224 Hartree**

0 1

**Zn**	**1.110613**	**-0.412906**	**0.208198**
**O**	**0.502467**	**1.394676**	**0.013256**
**H**	**1.002775**	**2.206172**	**-0.016524**
**O**	**2.616226**	**-1.363977**	**0.400512**
**H**	**2.528907**	**-2.316161**	**0.473379**
**Zn**	**-1.390403**	**1.115503**	**-0.106231**
**O**	**-0.799314**	**-0.719868**	**0.090132**
**H**	**-1.299059**	**-1.531841**	**0.120806**
**O**	**-2.922750**	**2.023246**	**-0.296793**
**H**	**-3.735872**	**1.515248**	**-0.316080**

Table S18

Structure: $Zn_3(OH)_6$ **-5793.1608123 Hartree**

0 1

Zn	-2.698720	0.535267	0.014461
O	-1.515907	-0.784314	-0.690275
H	-1.720281	-1.616365	-1.109388
O	-4.434499	0.980084	0.097805
H	-4.667592	1.721427	0.659761
Zn	0.188913	-0.072491	-0.045110
O	-1.024648	1.266612	0.671258
H	-0.823881	2.127891	1.028882
O	1.826690	0.589367	-0.887488
H	1.990095	1.391342	-1.377256
Zn	3.072080	-0.683510	-0.205168
O	1.466138	-1.364061	0.647630
H	1.300349	-2.200142	1. 076006
O	4.809224	-1.128514	-0.249112
H	5.093483	-1.832000	0.337417

Table S19

Structure: TS3-1 **-5793.1006848 Hartree**

0 1

Zn	-3.246439	-0.734135	0.036217
O	-1.644749	-1.519426	-0.654753
H	1.534740	-2.287156	-1.210082
O	-5.022518	-0.957799	0.074394
H	-5.539822	-0.304200	0.548541
Zn	-0.333801	-0.278300	0.023792
O	-1.931039	0.521324	0.730057
H	-2.035536	1.295195	1.278387
O	3.722382	2.619656	0.618425
H	4.580883	2.775383	0.218899
Zn	2.887936	1.128272	0.089526
O	1.433993	-0.051042	-0.027500
H	2.301692	-0.660395	-0.653649
O	3.460068	-0.501489	-1.015290
H	4.066641	-1.184673	-0.722579

Table S20

Structure: IM3-1 **-5793.1091240 Hartree**

0 1

Zn	-3.193601	0.098604	-0.049189
O	-1.869493	-1.278491	-0.100378
H	-1.973832	-2.219021	-0.219505
O	-4.964972	0.354026	-0.126329
H	-5.282535	1.257220	-0.071515
Zn	-0.260774	-0.225466	0.086097
O	-1.587355	1.175195	0.141476
H	-1.479230	2.115094	0.265092
O	4.472764	1.426193	-0.123421
H	5.265622	1.001729	-0.459029
Zn	3.056538	0.354119	-0.015862
O	1.492061	-0.487177	0.157210
H	2.551335	-1.812478	-0.042291
O	3.535947	-1.881520	-0.178994
H	3.909724	-2.429367	0.516160

Table S21

Structure: $Zn_3O_5H_4$ **-5716.6406833 Hartree**

0 1

Zn	3.171646	-0.096683	-0.000635
O	1.872839	1.308210	-0.067259
H	1.992772	2.253941	-0.095974
O	4.945322	-0.349305	0.036336
H	5.260445	-1.254374	0.070296
Zn	0.242049	0.290139	-0.060953
O	1.547816	-1.150452	0.007002
H	1.422953	-2.095908	0.035964
O	-4.408012	-1.482689	-0.074839
H	-5.256866	-1.038304	-0.122681
Zn	-2.954901	-0.472719	-0.086720
O	-1.513273	0.496224	-0.096548

Table S22

Structure: TS3-2 **-5716.6380686 Hartree**

0 1

Zn	-2.169941	-0.506891	0.057007
O	-1.603925	1.389645	0.031904
H	-2.133630	2.182922	0.041084
O	-3.851868	-1.117410	0.075743
H	-3.995511	-2.065159	0.083963
Zn	0.239736	1.195356	0.014183
O	-0.301043	-0.767702	0.052389
H	0.178834	-1.591767	-0.017267
O	3.689781	-1.722692	-0.078196
H	4.178709	-2.016249	0.693084
Zn	2.913648	-0.133320	-0.019639
O	2.012036	1.370918	-0.004767

Table S23

Structure: IM3-2 **-5716.6394670 Hartree**

0 1

Zn	-2.035682	-0.620962	0.025363
O	-1.724491	1.358925	-0.008315
H	-2.358977	2.071433	-0.003800
O	-3.694556	-1.290515	0.050724
H	-3.809688	-2.241921	0.030133
Zn	0.102003	1.425150	-0.016736
O	-0.161725	-0.694739	0.022326
H	0.327337	-1.520020	-0.069147
O	2.603639	-1.962429	-0.083723
H	2.894332	-2.417811	0.709237
Zn	2.363275	-0.198028	-0.019581
O	1.884485	1.506930	-0.013691

Table S24

Structure: TS3-3 **-5716.5832213 Hartree**

0 1

Zn	1.5011191	-0.957720	-0.349025
O	1. 872754	0.919919	-0.410420
H	3.081110	0.297058	-0.126687
O	3.448085	-0.758036	-0.035667
H	4.183734	-0.951035	-0.621730
Zn	0.258537	1.583740	-0.078763
O	-0.281889	-1.291571	-0.266567
H	-0.731302	-2.142165	-0.245367
O	-2.954553	-1.726595	0.208677
H	-3.706906	-1.872470	-0.367918
Zn	-2.091600	-0.142650	0.162929
O	-1.478560	1.545100	0.209768

Table S25

Structure: IM3-3 **-5716.5846338 Hartree**

0 1

**Zn**	**-1.513338**	**-0.901599**	**-0.017335**
**O**	**-1.946424**	**0.913229**	**0.027573**
**H**	**-3.409271**	**0.082284**	**-0.264020**
**O**	**-3.548882**	**-0.914600**	**-0.327482**
**H**	**-4.299466**	**-1.193281**	**0.203986**
**Zn**	**-0.293175**	**1.580332**	**0.015415**
**O**	**0.250294**	**-1.344966**	**0.060065**
**H**	**0.659889**	**-2.213994**	**0.038724**
**O**	**2.991598**	**-1.728375**	**-0.073994**
**H**	**3.690530**	**-1.892235**	**0.561919**
**Zn**	**2.077749**	**-0.173181**	**-0.011791**
**O**	**1.467650**	**1.518419**	**0.007214**

Table S26

Structure: $Zn_3O_4H_2$ **-5640.1052402 Hartree**

0 1

**Zn**	**1.354003**	**-0.674924**	**-0.061066**
**O**	**2.031270**	**0.984717**	**-0.031644**
**Zn**	**0.351437**	**1.692322**	**-0.008587**
**O**	**-0.269845**	**-1.445454**	**-0.074568**
**H**	**-0.532946**	**-2.366227**	**-0.079304**
**O**	**-3.116061**	**-1.688126**	**0.068627**
**H**	**-3.889196**	**-1.729944**	**-0.497298**
**Zn**	**-2.060278**	**-0.231869**	**0.010350**
**O**	**-1.393889**	**1.443250**	**-0.005470**

Table S27

Structure: TS3-4 **-5640.0695660 Hartree**

0 1

Zn	1.025649	1.387511	0.002465
O	2.387439	0.196480	-0.008924
Zn	1.270068	-1.228293	0.016906
O	-0.749979	1.374952	0.046282
H	-2.120614	1.535275	-0.176874
O	-2.992774	0.868852	-0.278874
H	-3.687803	1.080185	0.348540
Zn	-1.513047	-0.434122	-0.032931
O	-0.401598	-1.867944	0.028064

Table S28

Structure: IM3-4 **-5640.0699684 Hartree**

0 1

Zn	-0.831950	1.467668	-0.006997
O	-2.297742	0.405785	0.028276
Zn	-1.321975	-1.120495	0.005613
O	0.942096	1.344469	-0.0617148
H	2.513800	1.361262	0.101662
O	3.200808	0.596622	0.155855
H	3.902356	0.716550	-0.488919
Zn	1.491738	-0.500572	-0.005370
O	0.301974	-1.879018	-0.025504

Table S29

Structure: Zn_3O_3 **-5563.5895128 Hartree**

0 1

Zn	0.740046	1.484117	0.009177
O	2.232023	0.453196	-0.025164
Zn	1.431180	-1.174558	-0.015685
O	-1.069186	1.362501	0.045147
Zn	-1.218430	-0.445113	0.040711
O	-0.207805	-1.950607	0.014201

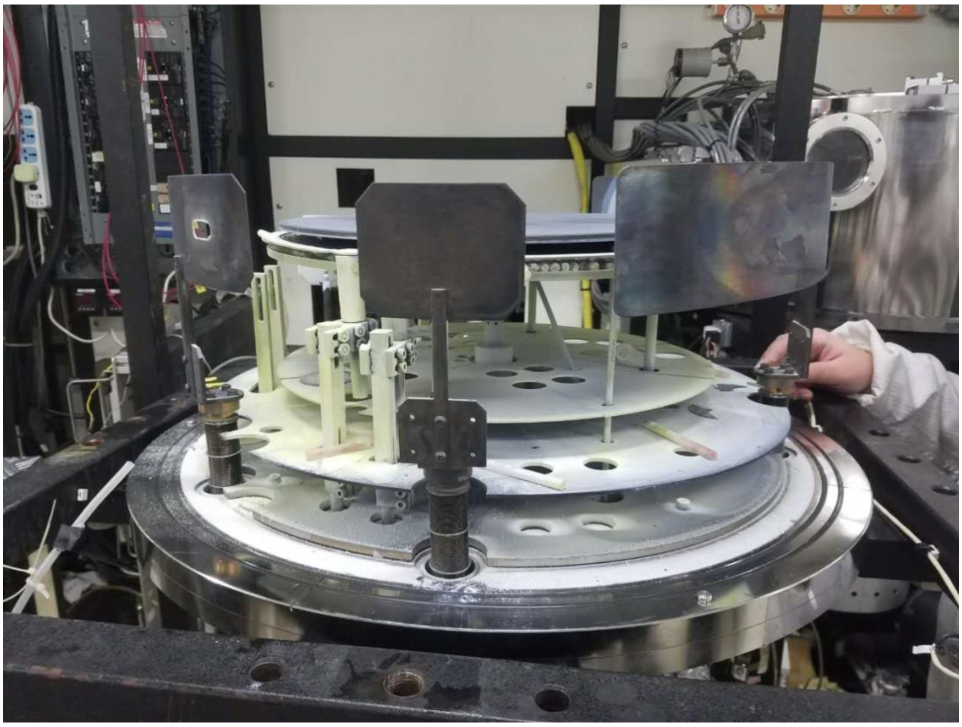

Fig. S1 Solid particles in the MOCVD reaction chamber

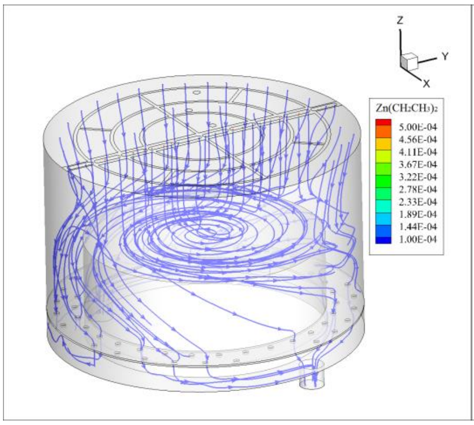
Z
Y
X
Zn(CH2CH3)2
5.00E-04
4.56E-04
4.11E-04
3.67E-04
3.22E-04
2.78E-04
2.33E-04
1.89E-04
1.44E-04
1.00E-04

(a)

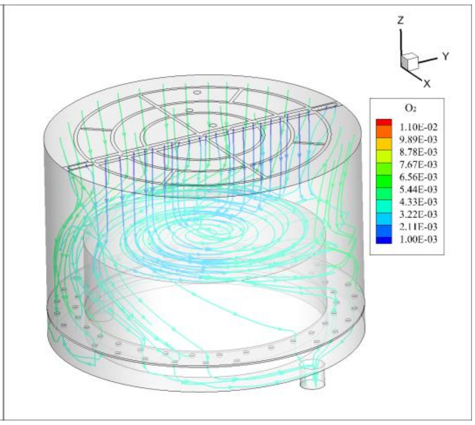
Z
Y
X
O2
1.10E-02
9.89E-03
8.78E-03
7.67E-03
6.56E-03
5.44E-03
4.33E-03
3.22E-03
2.11E-03
1.00E-03

(b)

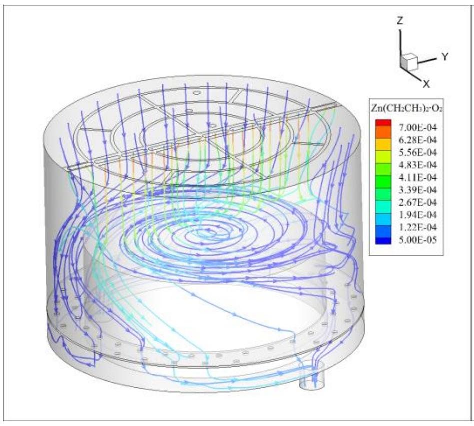
Z
Y
X
Zn(CH2CH3)2·O2
7.00E-04
6.28E-04
5.56E-04
4.83E-04
4.11E-04
3.39E-04
2.67E-04
1.94E-04
1.22E-04
5.00E-05

(c)

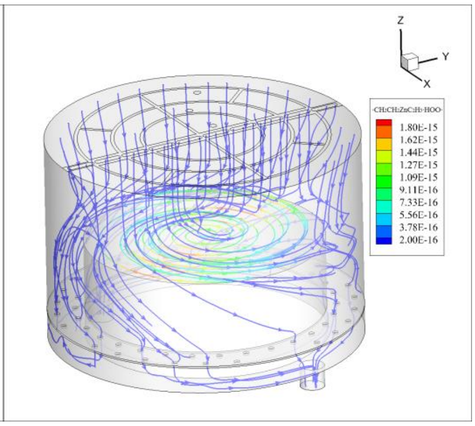
Z
Y
X
1.80E-15
1.62E-15
1.44E-15
1.27E-15
1.09E-15
9.11E-16
7.33E-16
5.56E-16
3.78E-16
2.00E-16

(d)

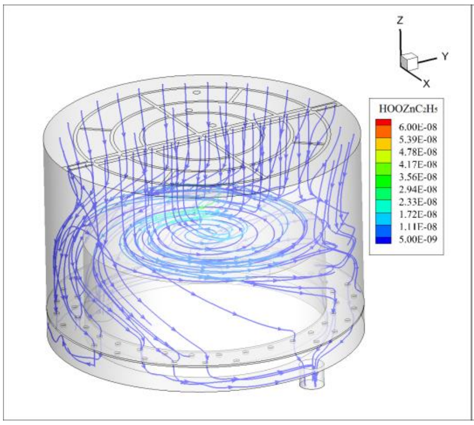
Z
Y
X
HOOZnC2H5
6.00E-08
5.39E-08
4.78E-08
4.17E-08
3.56E-08
2.94E-08
2.33E-08
1.72E-08
1.11E-08
5.00E-09

(e)

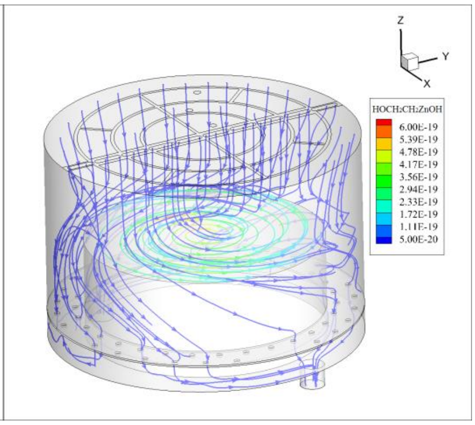
Z
Y
X
HOCH2CH2ZnOH
6.00E-19
5.39E-19
4.78E-19
4.17E-19
3.56E-19
2.94E-19
2.33E-19
1.72E-19
1.11E-19
5.00E-20

(f)

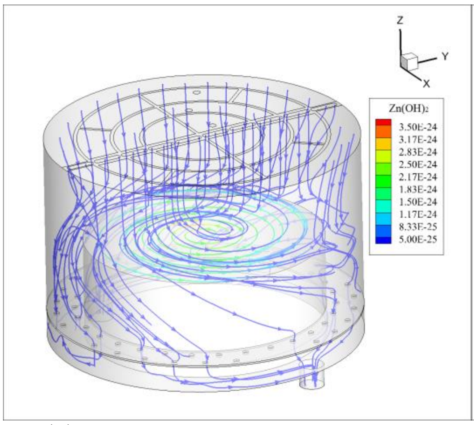
Z
Y
X
Zn(OH)2
3.50E-24
3.17E-24
2.83E-24
2.50E-24
2.17E-24
1.83E-24
1.50E-24
1.17E-24
8.33E-25
5.00E-25

(g)

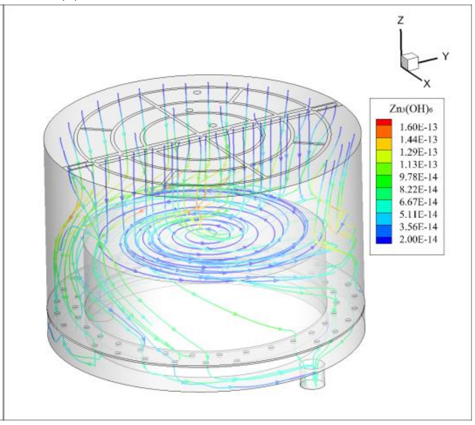
Z
Y
X
1.60E-13
1.44E-13
1.29E-13
1.13E-13
9.78E-14
8.22E-14
6.67E-14
5.11E-14
3.56E-14
2.00E-14

(h)

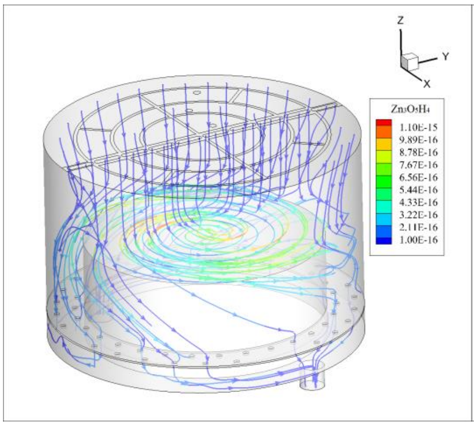

(i)

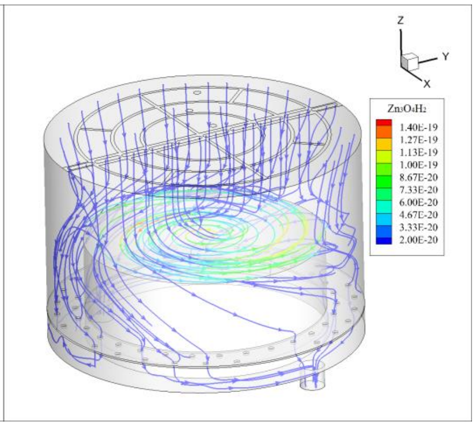

(j)

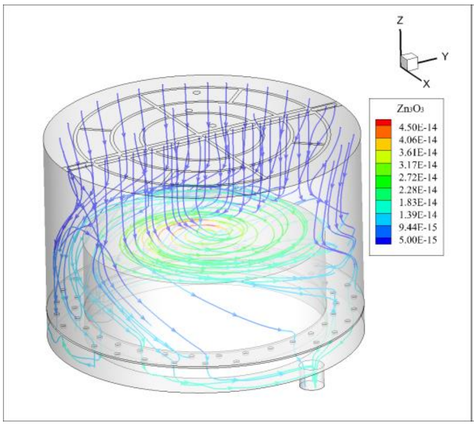

(k)

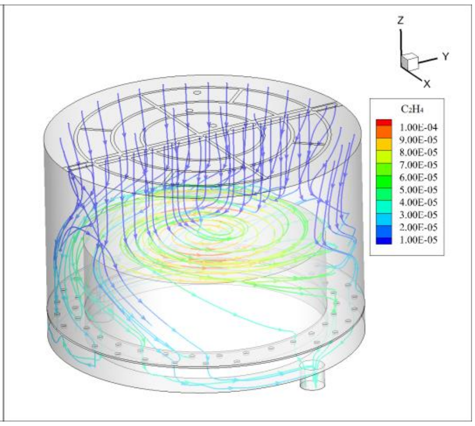

(l)

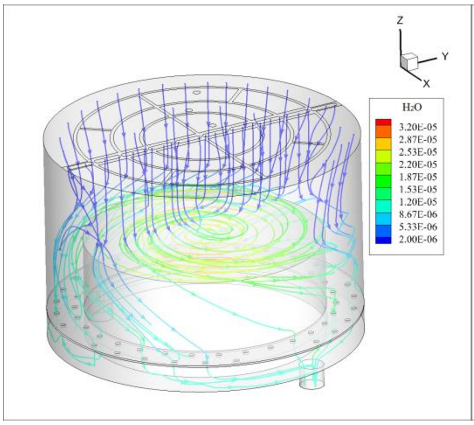

(m)

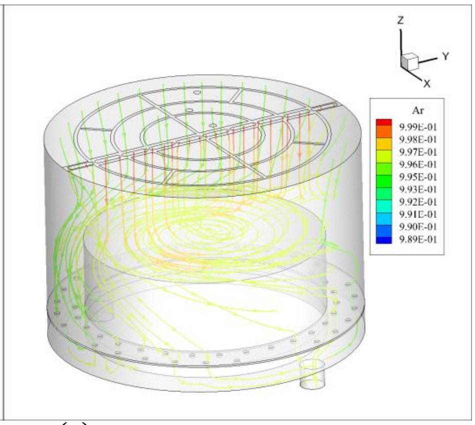

(n)

Fig. S2 The flows of the mass fractions of relevant species with the temperature of 773K: (a) $Zn(CH_2CH_3)_2$, (b) O_2, (c) $Zn(CH_2CH_3)_2$•O_2, (d) •$CH_2CH_2ZnC_2H_5$•HOO•, (e) $HOOZnC_2H_5$, (f) $HOCH_2CH_2ZnOH$, (g) $Zn(OH)_2$, (h) $Zn_3(OH)_6$, (i) $Zn_3O_5H_4$, (j) $Zn_3O_4H_2$, (k) Zn_3O_3. (l) C_2H_4, (m) H_2O, (n) Ar

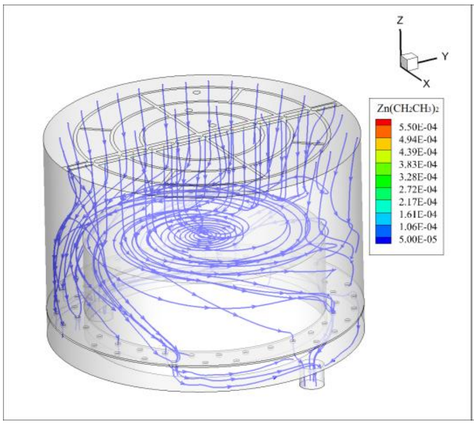
Z
Y
X
Zn(CH2CH3)2
5.50E-04
4.94E-04
4.39E-04
3.83E-04
3.28E-04
2.72E-04
2.17E-04
1.61E-04
1.06E-04
5.00E-05

(a)

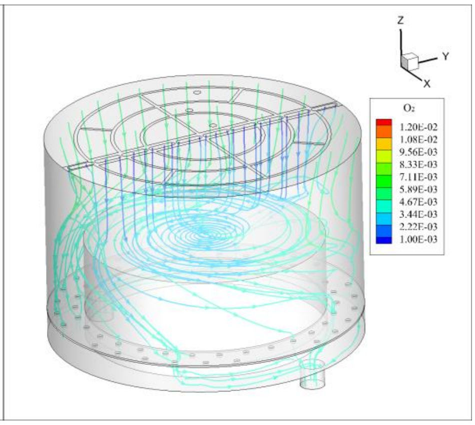
Z
Y
X
O2
1.20E-02
1.08E-02
9.56E-03
8.33E-03
7.11E-03
5.89E-03
4.67E-03
3.44E-03
2.22E-03
1.00E-03

(b)

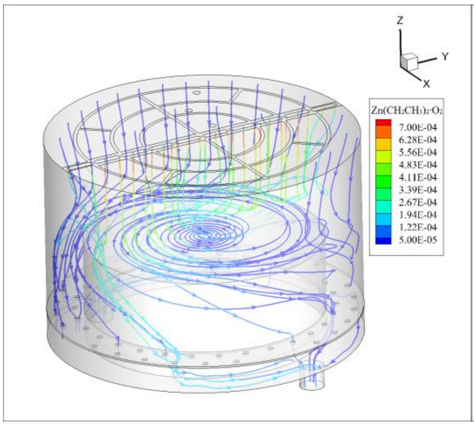
Z
Y
X
Zn(CH2CH3)2·O2
7.00E-04
6.28E-04
5.56E-04
4.83E-04
4.11E-04
3.39E-04
2.67E-04
1.94E-04
1.22E-04
5.00E-05

(c)

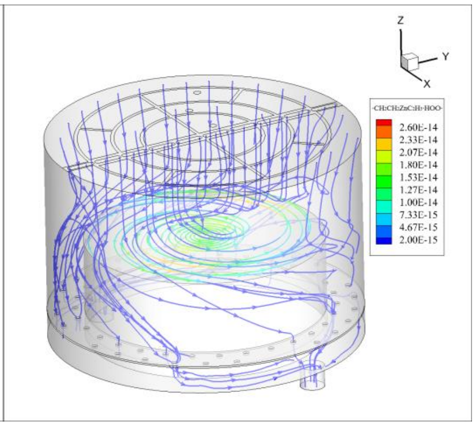
Z
Y
X
·CH2CH2ZnC2H5·HOO·
2.60E-14
2.33E-14
2.07E-14
1.80E-14
1.53E-14
1.27E-14
1.00E-14
7.33E-15
4.67E-15
2.00E-15

(d)

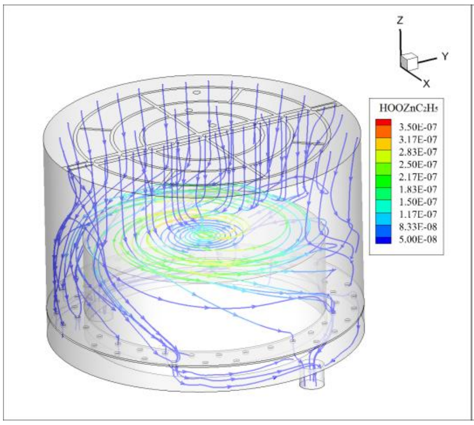
Z
Y
X
HOOZnC2H5
3.50E-07
3.17E-07
2.83E-07
2.50E-07
2.17E-07
1.83E-07
1.50E-07
1.17E-07
8.33E-08
5.00E-08

(e)

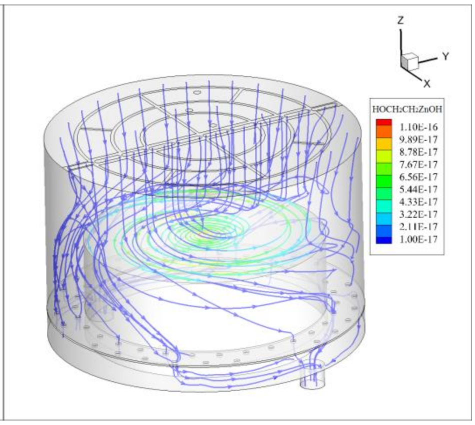
Z
Y
X
HOCH2CH2ZnOH
1.10E-16
9.89E-17
8.78E-17
7.67E-17
6.56E-17
5.44E-17
4.33E-17
3.22E-17
2.11E-17
1.00E-17

(f)

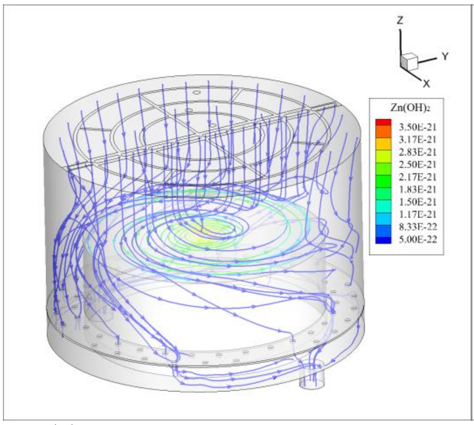
Z
Y
X
Zn(OH)2
3.50E-21
3.17E-21
2.83E-21
2.50E-21
2.17E-21
1.83E-21
1.50E-21
1.17E-21
8.33E-22
5.00E-22

(g)

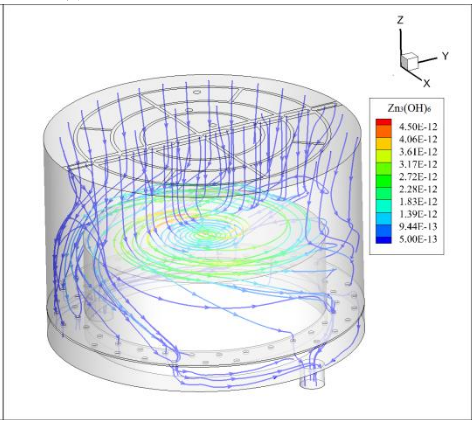
Z
Y
X
Zn3(OH)6
4.50E-12
4.06E-12
3.61E-12
3.17E-12
2.72E-12
2.28E-12
1.83E-12
1.39E-12
9.44E-13
5.00E-13

(h)

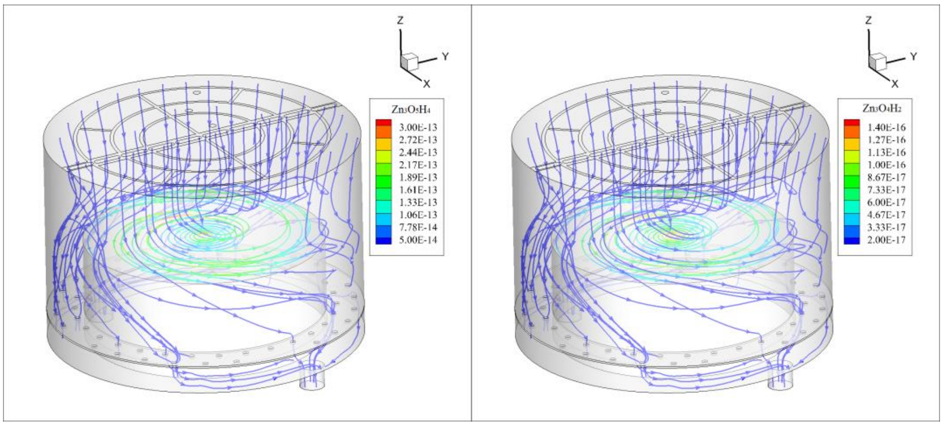

(i) (j)

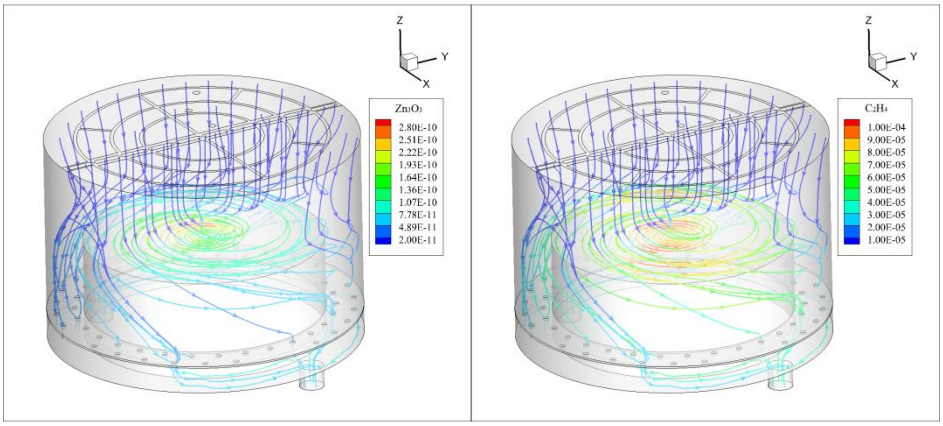

(k) (l)

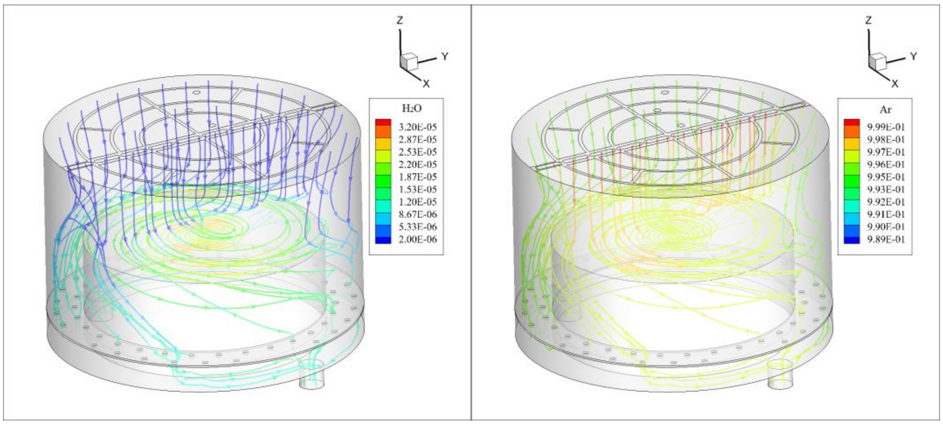

(m) (n)

Fig. S3 The flows of the mass fractions of relevant species with the temperature of 873K: (a) $Zn(CH_2CH_3)_2$, (b) O_2, (c) $Zn(CH_2CH_3)_2$•O_2, (d) •$CH_2CH_2ZnC_2H_5$•HOO•, (e) $HOOZnC_2H_5$, (f) $HOCH_2CH_2ZnOH$, (g) $Zn(OH)_2$, (h) $Zn_3(OH)_6$, (i) $Zn_3O_5H_4$, (j) $Zn_3O_4H_2$, (k) Zn_3O_3. (l) C_2H_4, (m) H_2O, (n) Ar

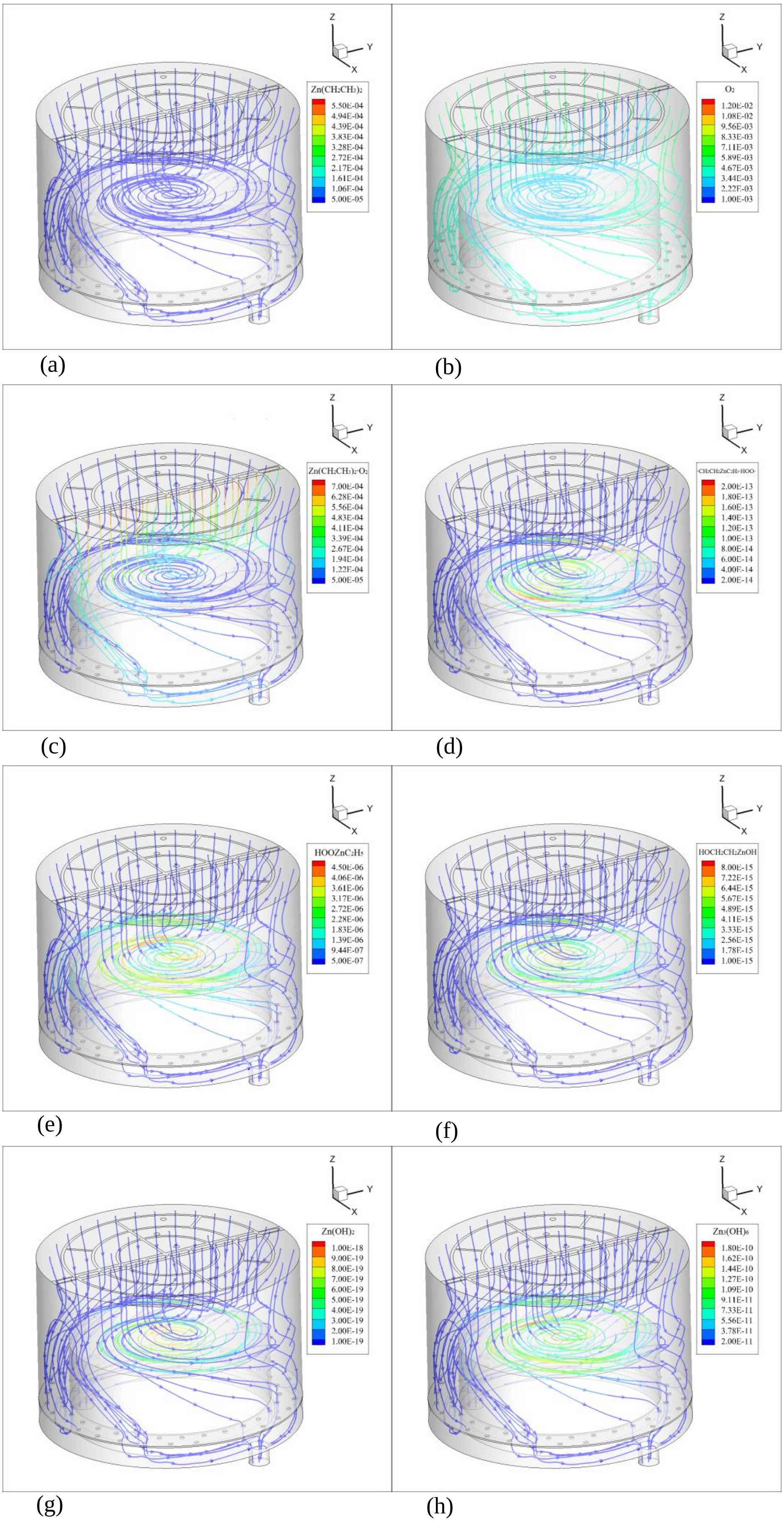
Z
Y
X
Zn(CH2CH3)2
5.50E-04
4.94E-04
4.39E-04
3.83E-04
3.28E-04
2.72E-04
2.17E-04
1.61E-04
1.06E-04
5.00E-05
O2
1.20E-02
1.08E-02
9.56E-03
8.33E-03
7.11E-03
5.89E-03
4.67E-03
3.44E-03
2.22E-03
1.00E-03
Zn(CH2CH3)2·O2
7.00E-04
6.28E-04
5.56E-04
4.83E-04
4.11E-04
3.39E-04
2.67E-04
1.94E-04
1.22E-04
5.00E-05
2.00E-13
1.80E-13
1.60E-13
1.40E-13
1.20E-13
1.00E-13
8.00E-14
6.00E-14
4.00E-14
2.00E-14
HOOZnC2H5
4.50E-06
4.06E-06
3.61E-06
3.17E-06
2.72E-06
2.28E-06
1.83E-06
1.39E-06
9.44E-07
5.00E-07
HOCH2CH2ZnOH
8.00E-15
7.22E-15
6.44E-15
5.67E-15
4.89E-15
4.11E-15
3.33E-15
2.56E-15
1.78E-15
1.00E-15
Zn(OH)2
1.00E-18
9.00E-19
8.00E-19
7.00E-19
6.00E-19
5.00E-19
4.00E-19
3.00E-19
2.00E-19
1.00E-19
Zn3(OH)6
1.80E-10
1.62E-10
1.44E-10
1.27E-10
1.09E-10
9.11E-11
7.33E-11
5.56E-11
3.78E-11
2.00E-11
(a)
(b)
(c)
(d)
(e)
(f)
(g)
(h)

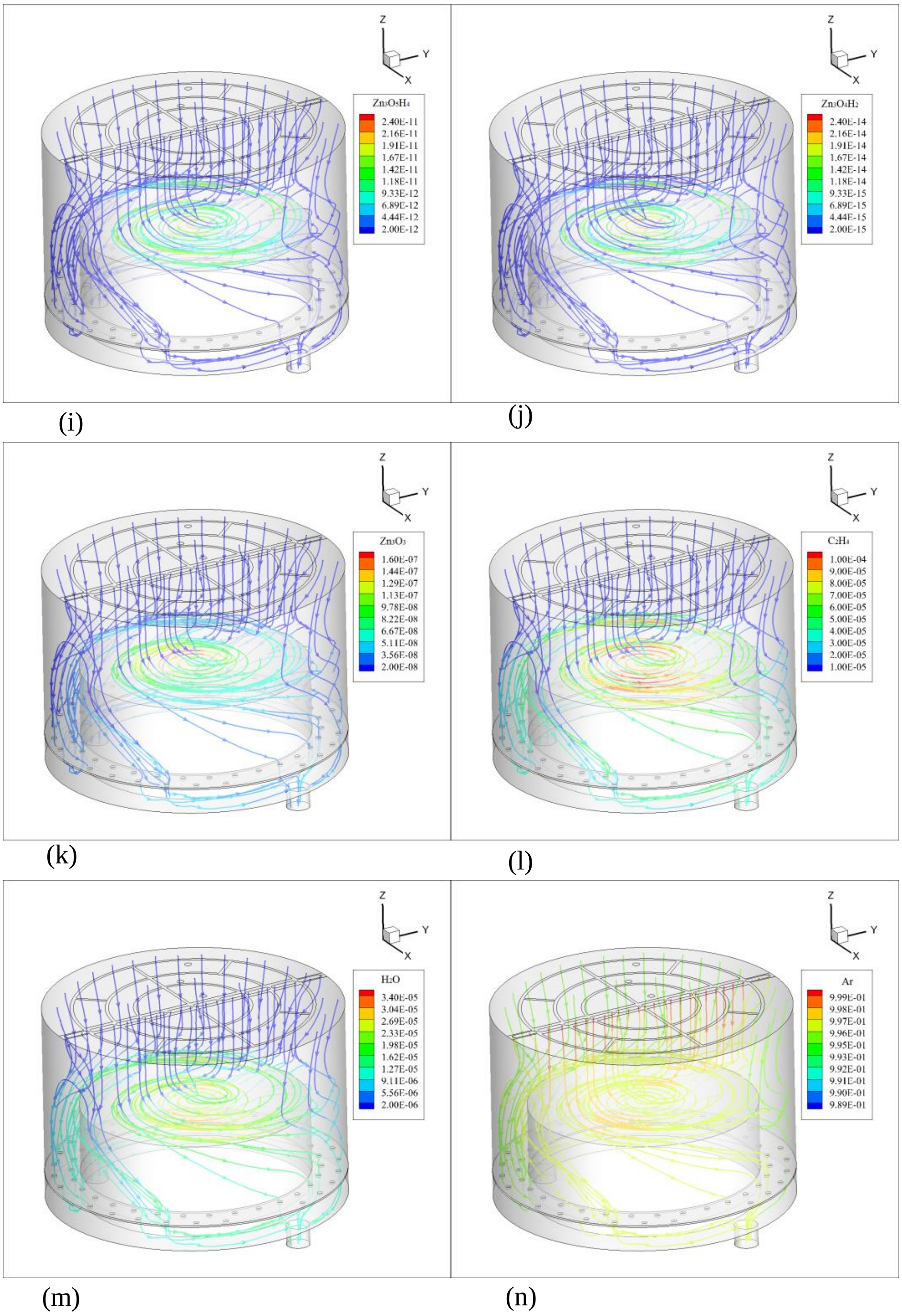

Fig. S4 The flows of the mass fractions of relevant species with the temperature of 973K: (a) $Zn(CH_2CH_3)_2$, (b) O_2, (c) $Zn(CH_2CH_3)_2$•O_2, (d) •$CH_2CH_2ZnC_2H_5$•HOO•, (e) $HOOZnC_2H_5$, (f) $HOCH_2CH_2ZnOH$, (g) $Zn(OH)_2$, (h) $Zn_3(OH)_6$, (i) $Zn_3O_5H_4$, (j) $Zn_3O_4H_2$, (k) Zn_3O_3. (l) C_2H_4, (m) H_2O, (n) Ar

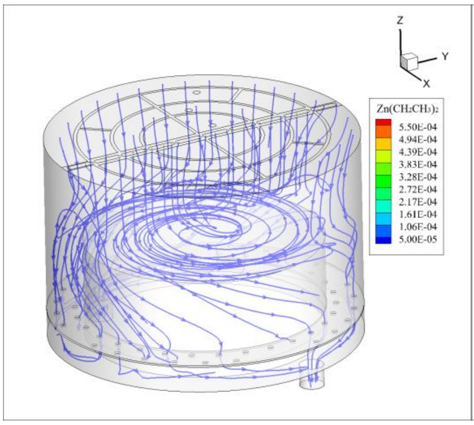

Z
Y
X
Zn(CH2CH3)2
5.50E-04
4.94E-04
4.39E-04
3.83E-04
3.28E-04
2.72E-04
2.17E-04
1.61E-04
1.06E-04
5.00E-05

(a)

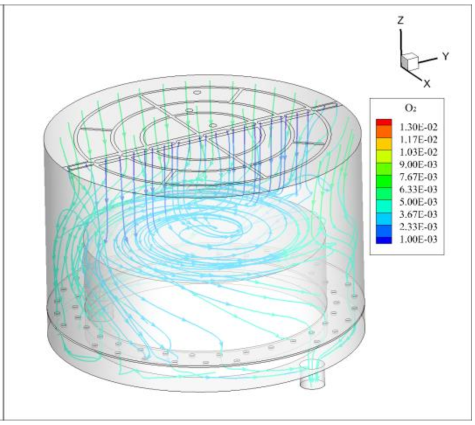

Z
Y
X
O2
1.30E-02
1.17E-02
1.03E-02
9.00E-03
7.67E-03
6.33E-03
5.00E-03
3.67E-03
2.33E-03
1.00E-03

(b)

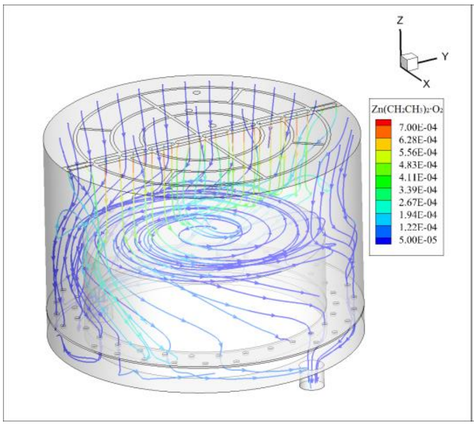

Z
Y
X
Zn(CH2CH3)2·O2
7.00E-04
6.28E-04
5.56E-04
4.83E-04
4.11E-04
3.39E-04
2.67E-04
1.94E-04
1.22E-04
5.00E-05

(c)

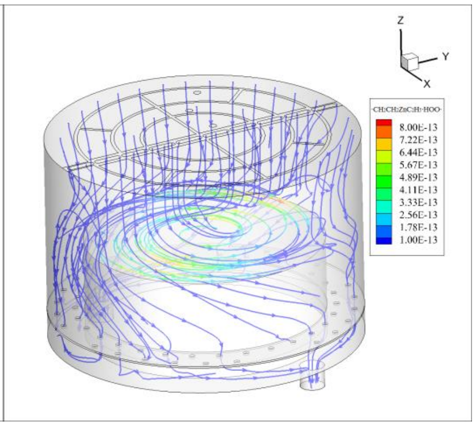

Z
Y
X
·CH2CH2ZnC2H5·HOO·
8.00E-13
7.22E-13
6.44E-13
5.67E-13
4.89E-13
4.11E-13
3.33E-13
2.56E-13
1.78E-13
1.00E-13

(d)

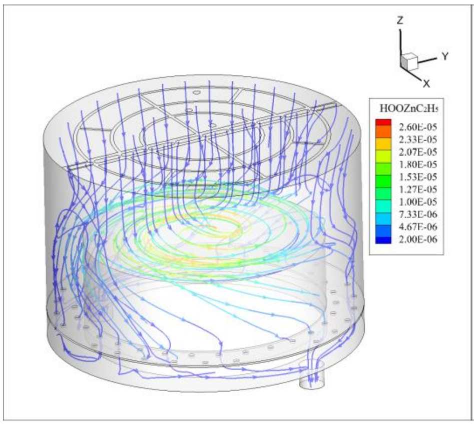

Z
Y
X
HOOZnC2H5
2.60E-05
2.33E-05
2.07E-05
1.80E-05
1.53E-05
1.27E-05
1.00E-05
7.33E-06
4.67E-06
2.00E-06

(e)

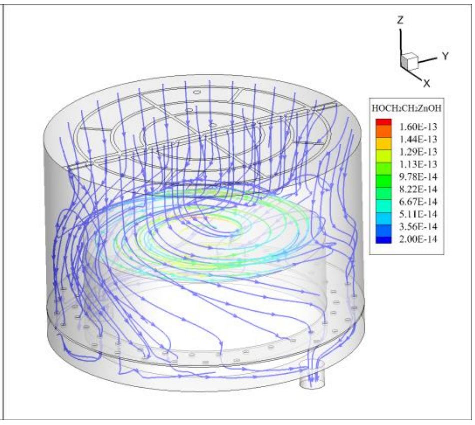

Z
Y
X
HOCH2CH2ZnOH
1.60E-13
1.44E-13
1.29E-13
1.13E-13
9.78E-14
8.22E-14
6.67E-14
5.11E-14
3.56E-14
2.00E-14

(f)

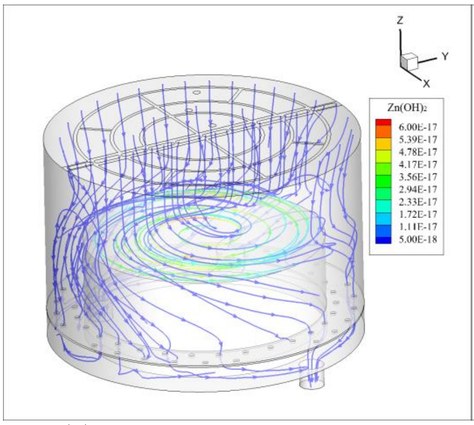

Z
Y
X
Zn(OH)2
6.00E-17
5.39E-17
4.78E-17
4.17E-17
3.56E-17
2.94E-17
2.33E-17
1.72E-17
1.11E-17
5.00E-18

(g)

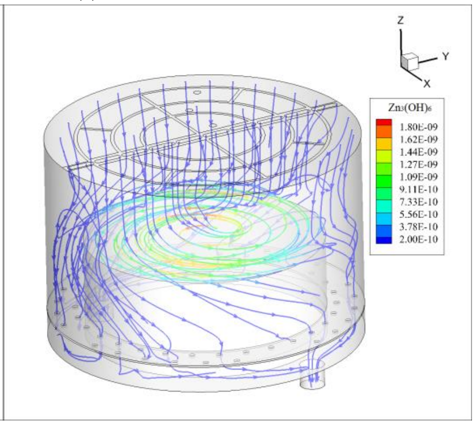

Z
Y
X
Zn3(OH)6
1.80E-09
1.62E-09
1.44E-09
1.27E-09
1.09E-09
9.11E-10
7.33E-10
5.56E-10
3.78E-10
2.00E-10

(h)

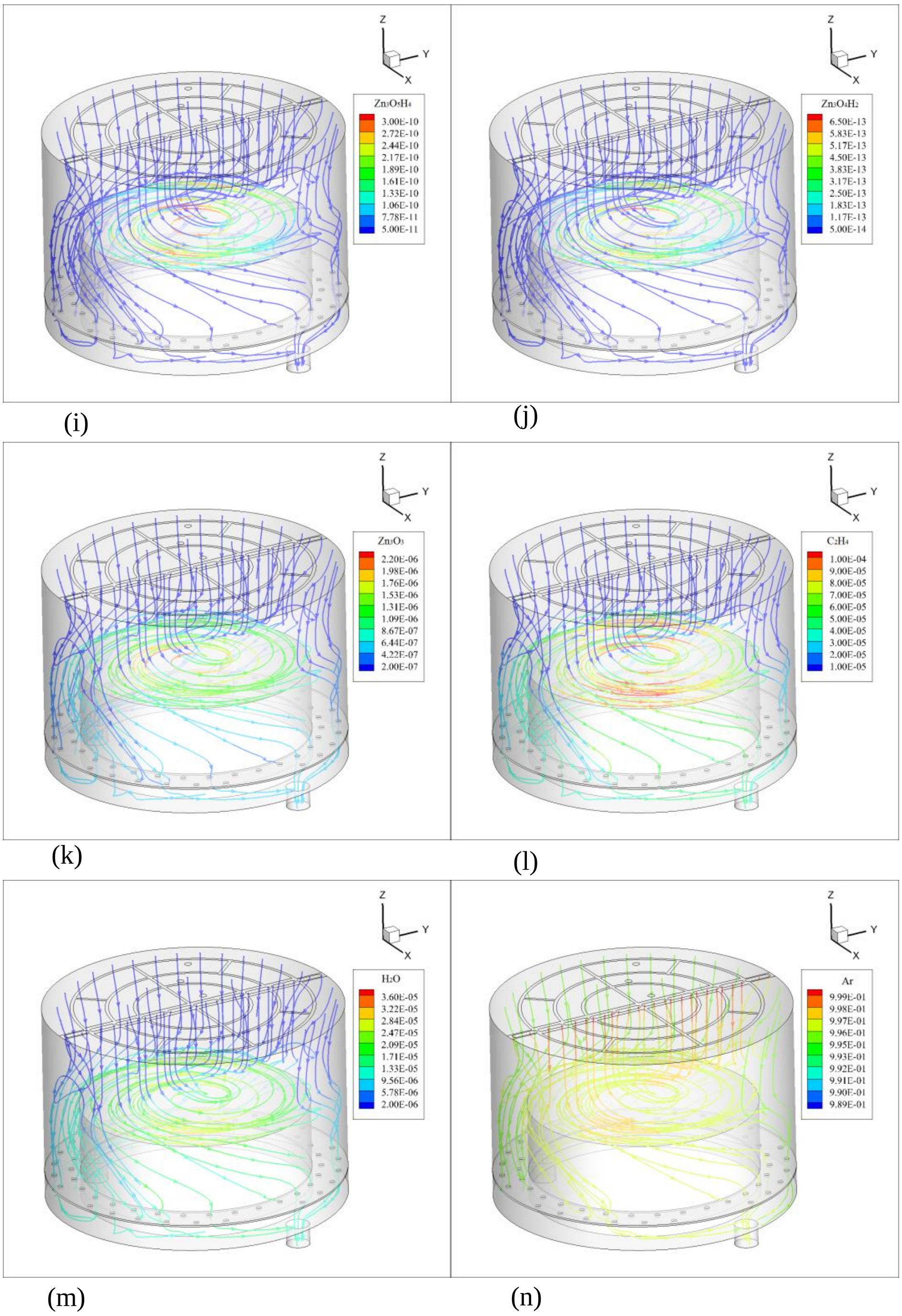

Fig. S5 The flows of the mass fractions of relevant species with the temperature of 1073K: (a) $Zn(CH_2CH_3)_2$, (b) O_2, (c) $Zn(CH_2CH_3)_2$•O_2, (d) •$CH_2CH_2ZnC_2H_5$•HOO•, (e) $HOOZnC_2H_5$, (f) $HOCH_2CH_2ZnOH$, (g) $Zn(OH)_2$, (h) $Zn_3(OH)_6$, (i) $Zn_3O_5H_4$, (j) $Zn_3O_4H_2$, (k) Zn_3O_3. (l) C_2H_4, (m) H_2O, (n) Ar
